# A Green Chemo-Enzymatic Approach for CO_2_ Capture and Transformation into Bis(cyclic carbonate) Esters in Solvent-Free Media

**DOI:** 10.1021/acssuschemeng.4c04102

**Published:** 2024-10-02

**Authors:** Rocio Villa, Francisco J. Ruiz, Francisco Velasco, Susana Nieto, Raul Porcar, Eduardo Garcia-Verdugo, Pedro Lozano

**Affiliations:** † Departamento de Bioquimica y Biologia Molecular B e Inmunologia. Facultad de Quimica, Universidad de Murcia, E-30100 Murcia, Spain; ‡ Departamento de Quimica Organica e Inorganica, Universidad Jaime I, E-12071 Castellon, Spain; § Departamento de Química Orgánica y Bio-orgánica, Facultad de Ciencias, Universidad Nacional de Educación a Distancia, UNED, Avda. Esparta, 28232 Las Rozas, Madrid, Spain

**Keywords:** CO_2_ capture, biocatalysis, supported ionic liquids, cyclic carbonates, sustainable chemistry, chemo-enzymatic process

## Abstract

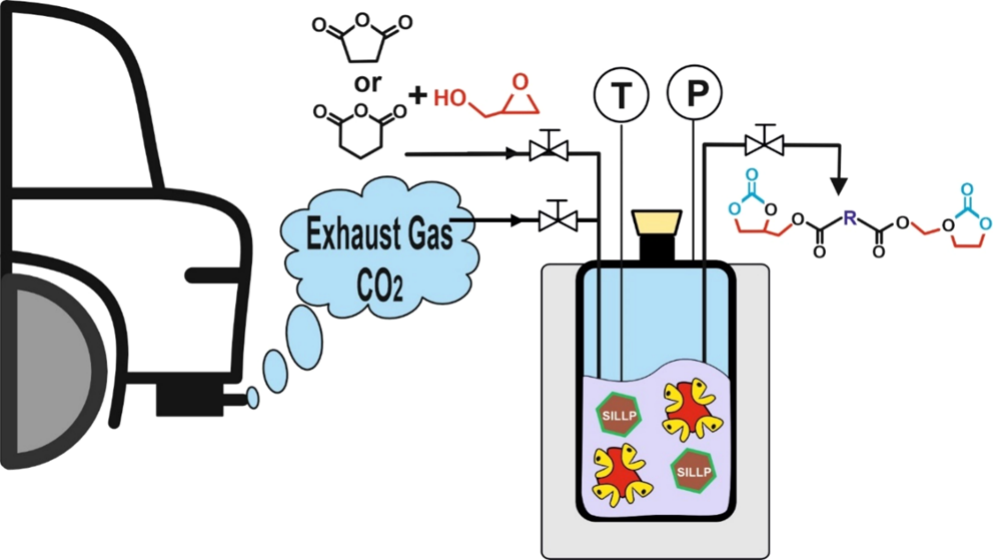

A sustainable approach for CO_2_ capture and chemo-enzymatic transformation into bis(cyclic carbonate) esters from CO_2_, glycidol, and organic anhydrides under solvent-free conditions has been demonstrated. The chemo-enzymatic process is based on two consecutive catalytic steps, which can be executed through separated operations or within a one-pot combo system, taking advantage of the synergic effects that emerge from integrating ionic liquid (IL) technologies and biocatalysts. In a first step, lipase-catalyzed transesterification and esterification reactions of different diacyl donors (e.g., glutaric anhydride, succinic anhydride, dimethyl succinate, etc.) with glycidol in solvent-free under mild reaction conditions (70 °C, 6 h) produce the corresponding diglycidyl ester derivatives in up to 41% yield. By a second step, the synthesis of bis(cyclic carbonate) esters was carried out as a result of the cycloaddition reaction of CO_2_ (from an exhausted gas source, 15% CO_2_ purity) on these diglycidyl esters, catalyzed by the covalently attached 1-decyl-2-methylimidazolium IL (supported ionic liquid-like phase, SILLP), in solvent-free condition, leading up to 65% yield after 8 h at 45 °C and 1 MPa CO_2_ pressure. Both key elements of the reaction system (biocatalyst and SILLP) were successfully recovered and reused for at least 5 operational cycles. Finally, different metrics have been applied to assess the greenness of the solvent-free chemo-enzymatic synthesis of bis(cyclic carbonate) esters here reported.

## Abstract 2

Green chemo-enzymatic approaches for CO_2_ capture from an exhaust gas, and its transformation into bis(cyclic carbonate) esters provide suitable compounds of high interest for the polymer industry.

## Introduction

Our life activity on the planet inevitably runs essentially through oxidative processes with the generation of CO_2_ as the ultimate universal waste product. The emission of CO_2_ into the atmosphere, along with the industrial production of recalcitrant plastic materials that are typically disposed of in landfilled or incinerated, remains to be among the most impacting wastes causing environmental damages.^1^ Ionic liquids (ILs) are widely recognized as efficient tools for any type of catalytic process,^2,3^ being in the core of new clean approaches for capture and transformation of CO_2_ into added-value products (e.g., methanol,^4^ formic acid,^5^ dimethyl carbonate,^6,7^ etc.). Among various direct (bio)catalytic approaches for transforming CO_2_ into valuable chemicals,^8^ the synthesis of cyclic organic carbonates stands out as a prominent focus within the scope of nonreductive CO_2_ transformations.^9^ Indeed, by using biosourced feedstock, such as epoxidized terpenes or vegetable oils, it is possible to produce biobased (poly)cyclic carbonates. This opens up avenues for creating a broad range of new sustainable products with several applications, in which the CO_2_ molecule is incorporated into their structures. Examples include the production of drop-in monomers for the preparation of sustainable polymers, like nonisocyanate polyurethanes (NIPUs) through aminolysis reactions with biobased polyamines,^10^ as well as elastic rubbers from CO_2_-based polycarbonates.^11^ In this scenario, employing glycidol or glycerol carbonate as feedstock is particularly compelling because both can be considered biobased materials derived from glycerol, which is the primary byproduct from the industrial biodiesel synthesis, being produced in quantities exceeding its current consumption.^12^ Glycidol preparation from biomass, instead of its fossil-based production, and its further conversion to value-added products could represent viable opportunities to boost the glycerol biorefinery.^13,14^ The presence of hydroxyl, epoxy, or cyclic carbonate groups enables the preparation of useful monomers for polymer synthesis, even permitting the easy introduction of other reactive moieties (e.g., acrylate groups) to obtain new functionalized monomers.^1,15^ Given these characteristics, focusing on the production of glycidol and glycerol carbonate derivatives represents an exciting and meaningful goal for chemists, promising advancements in sustainable chemical processes and materials.^16,17^ In the same way, biobased bis(cyclic carbonate) compounds, obtained from feedstock materials (e.g., sorbitol isosorbide, unsaturated acids, limonene, lysine, glycerol carbonate derivatives, etc.), are of special interest for the preparation of polyesters, polycarbonates, and NIPUs.^18−21^ However, the efficiency (e.g., up to 45% yield after 48 h at 75 °C^22^) and the greenness (e.g., the use of toxic epichlorohydrin and volatile organic solvents,^23^) of these processes can greatly be improved. As a representative example, it was reported the synthesis of the biobased bis(cyclic carbonate) succinate by the direct esterification of succinic acid with glycerol carbonate, using *p*-toluenesulfonic acid as a catalyst, in toluene heated to reflux for 5 h. An 85% bis(cyclic carbonate) product yield was obtained after several separation steps with other organic solvents.^24^ Alternatively, the biocatalytic synthesis of sebacic bis(cyclic carbonate) has been developed by means of the direct esterification of sebacic acid with glycerol carbonate at a 1:100 mol/mol ratio, using a drying agent (Na_2_SO_4_) and at reduced pressure (200 mbar) to eliminate the released water. By this strategy, a product yield up to 100% was obtained after incubation for 3 days at room temperature.^25^ Another interesting approach for the synthesis of biobased cyclic carbonates and polycarbonates consisted of the use of unpurified CO_2_ as substrate obtained from sugar fermentation and, directly applied for the cycloaddition reaction into different epoxides (e.g. eugenol oxide, styrene oxide, etc.) using a Ga-catalyst, achieving yields up to 100% under mild reaction conditions after 24 h.^26^

Also, it should be noted that the synthesis of glycerol carbonate from glycerol and CO_2_ is not an easy task because of the necessity of using stoichiometric amounts of dehydrating agents (e.g., calcium carbide, CaC_2_) to shift the process to the product side, as well as the harsh reaction conditions (i.e. 50 bar CO_2_ and 180 °C for 24 h), which finally resulted in up to 92% yield (isolated 88%).^4,27^ Therefore, polymers derived from natural compounds and synthesized by greener catalysts and reaction media are highly desirable.

Among other criteria, the greenness of chemical processes is strongly sustained by several pillars, such as the selectivity catalytic transformations, the use of biobased feedstock, as well as by reducing, or avoiding, the use of volatile organic solvents.^28^ Enzymes constitute the most powerful toolbox of catalysts for performing selective, clean, and sustainable transformations, mainly when using them in nonaqueous environments. Lipases are by far the most widely used biocatalysts in green reaction media (e.g., ILs, supercritical fluids, deep eutectic mixtures, etc.) at low water content and, as such, they are used for the synthesis of aliphatic and aromatic esters, chiral esters by kinetic resolution of racemic alcohols, carbohydrate esters, and even polymers.^29^ Novozym 435 was previously described as a suitable biocatalyst to carry out direct esterification reactions between carboxylic acids and alcohols (e.g., lauric acid and panthenol,^30^ decanoic acid and xylitol,^31^ etc.) in solvent-free and deep eutectic solvents (DESs)-like reaction media of high viscosity. Also, lipase-catalyzed ring-opening polymerization of biocompound-based cyclic monomers (e.g., carbonate-based macrocycles, lactones, cyclic anhydrides, etc.) has emerged as a promising and green strategy for the design and synthesis of such polymers, giving a more precise construction of well-defined structures than conventional chemical catalysis.^32^ In the same context, supported ionic liquid-like phases (SILLPs) have emerged as highly effective catalysts for the cycloaddition of CO_2_ to epoxides under relatively low CO_2_ pressures (0.1–1 MPa) and moderate temperatures (60 °C), maintaining a high activity and stability level for 10 days in a flow system.^33^ The combination of this SILLP-based catalyst with an immobilized lipase has been successfully applied to the synthesis of glycerol carbonate (meth)acrylate through the cycloaddition of CO_2_ to glycidyl (meth)acrylate, showcasing the potential of these catalysts in facilitating green chemical processes.^15^

**Figure 1:**
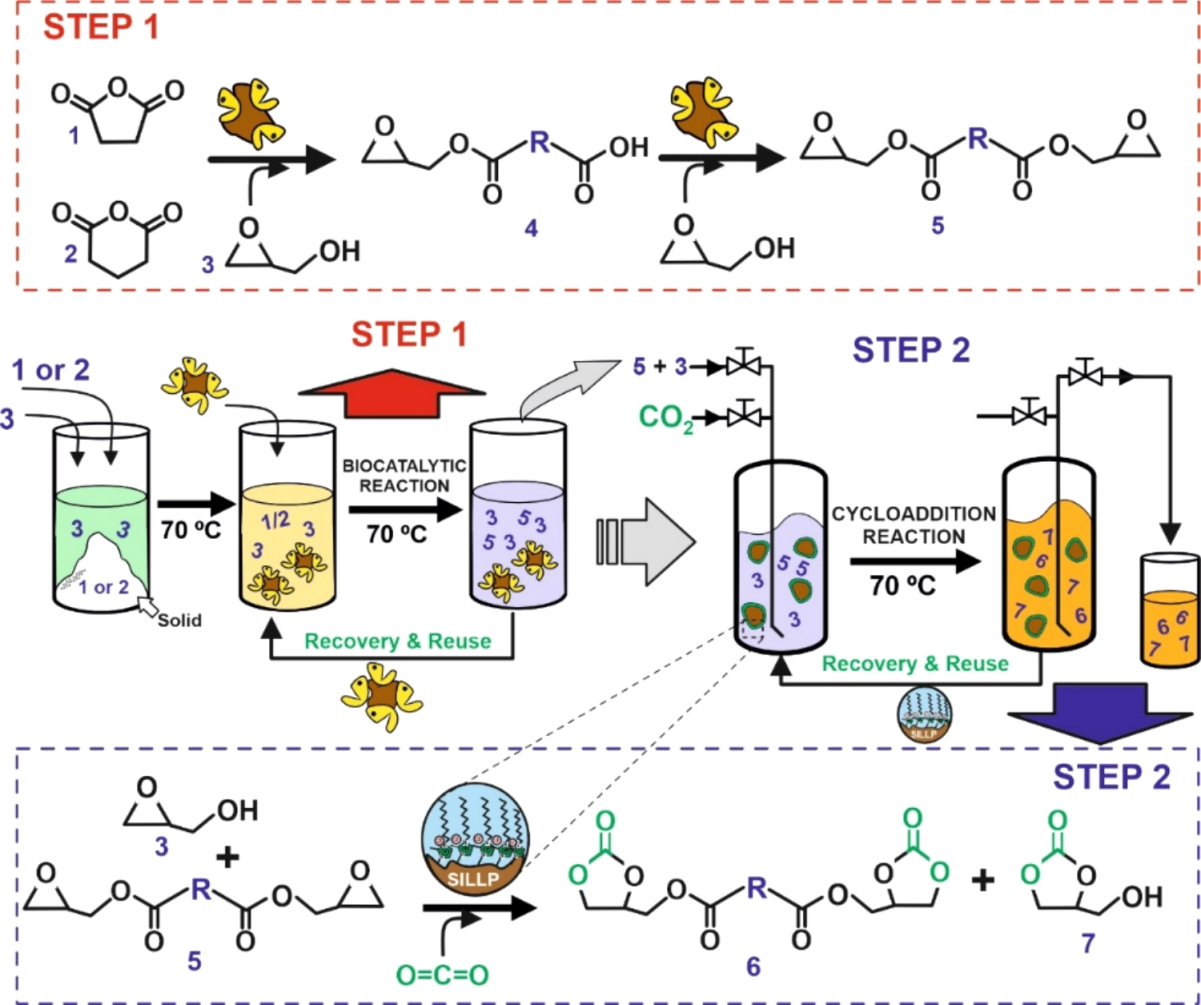
Chemo-enzymatic synthesis of bis(cyclic carbonate)s by means of two consecutive steps. Step 1. Novozym 435 catalyzes the synthesis of diglycidyl succinate (or glutarate) (**5**) by ring opening of succinic anhydride (SA, **1**) or glutaric anhydride (GA, **2**), with glycidol (**3**) in solvent-free. Step 2. 1-Alkyl-3-methylimidazolium-based SILLP-catalyzed synthesis of bis(cyclic carbonate)s succinate or glutarate (**6**) through the cycloaddition of CO_2_ into the epoxide ring of (**5**). **R** = −CH_2_–, (SA); or −CH_2_–CH_2_–, (GA).

This work presents, for the first time, the synthesis of biobased bis(cyclic carbonate) succinate or glutarate using a two-step catalytic method by using cyclic anhydrides, glycidol, and CO_2_ as substrates. This approach capitalizes on the synergistic interaction between biocatalysis and supported IL-based technologies in solvent-free environments (Figure 1). The first step runs through a lipase-catalyzed ring-opening reaction of the corresponding cyclic anhydride, followed by double esterification with 1-glycidol that provides the corresponding succinate (or glutarate) diglycidyl ester in solvent-free media. By a second step, the synthesis of the corresponding bis(cyclic carbonate)s occurred as a result of a SILLPs-catalyzed CO_2_ cycloaddition reaction into the epoxide moieties of succinate (or glutarate) diglycidyl esters, providing the bis(cyclic carbonate)s as valuable monomers for the synthesis of NIPUs.^34^ The suitability of the proposed synthetic approach was successfully demonstrated by a two-step approach, as well as a one-pot-combo reaction system, even by using a synthetic exhaust gas as the CO_2_ source to highlight the environmental friendliness of this technology. Furthermore, the sustainability of these proposed chemo-enzymatic approaches for bis(cyclic carbonate) esters synthesis has been demonstrated through several green metric parameters.

## Experimental Section

### Chemicals

The commercial immobilized lipases Novozym 435 (*Candida antarctica* Lipase B, N-435), Lypozyme TL IM (*Thermomyces lanuginosus* Lipase), and Lypozyme RM IM (*Rhizomucor miehei* Lipase) were a gift from Novozymes S.A. (Spain). Glutaric anhydride (GA, 95% purity), succinic anhydride (SA, >99% purity), glutaric acid (99% purity, GAc), succinic acid (>99.5% purity, SAc), dimethyl glutarate (98% purity, DMG), dimethyl succinate (98% purity, DMS), glycidol (96% purity), glycerol carbonate (GC), molecular sieves 13X (MS 13X; adsorption capacity: 270 mg H_2_O/g), Merrifield resins (HL and LL, 100–200 mesh) reagents, solvents, and other chemicals were acquired from Merck-Sigma (Spain). Both pure CO_2_ (99.9999% purity) and exhaust gas containing N_2_ (84.0% w/w), CO_2_ (15.2% w/w), CO (0.4% w/w), and CH_4_ (0.4% w/w), respectively, were obtained from Nippon Gases (Spain).

### Preparation of SILLPs

Polymeric supports containing covalently attached alkyl-imidazolium moieties were prepared by chemical modification of commercially available chloromethylated Merrifield-gel polymers, as it was previously reported, which can be obtained with different counteranion by simple metathesis.^15,33^ All of these materials have been characterized by various techniques, such as Fourier transform infrared (FT-IR) spectroscopy or Raman microspectroscopy, which confirmed the expected structures.^33,35^

### Novozym 435-Catalyzed Synthesis of Diglycidyl Ester in Solvent-Free

Reaction mixtures were prepared in 4 mL vials by 1 or 2 mmol of acyl donor (i.e., glutaric anhydride, GA; glutaric acid, GAc; dimethyl glutarate, DMG, succinic anhydride, SA; succinic acid, SAc; or dimethyl succinate, DMS, respectively) with the corresponding amount of glycidol to substrate solutions at 1:9 and 1:7.5 molar ratio, resulting in fully clear solutions after shaken for 10 min at room temperature. For reaction media based on 1:6, 1:4, and 2:4.5 acyl donor:glycidol molar ratios, the mixture was preincubated for 10 min at 80 °C under magnetic stirring, leading to monophasic viscous media system in all cases (see Figures S5 and S6 for GC analysis of the mixture after the preheated treatment). For each scenario, the reaction was started by adding to the mixture the corresponding amount of biocatalyst (i.e., Novozym 435) and 50 mg of drying agent (i.e., MS 13X, MgSO_4_, or CaCl_2_ previously activated by microwaves for 1 min at 800 W) and was maintained under continuous shaking for the desired time at 50, 60, or 70 °C (IKA KS 4000 Control). The MS 13X molecular sieves were selected against other classical dehydrating agents (i.e., anhydrous MgSO_4_ or CaCl_2_) due to their better suitability for the proposed biocatalytic process (see Supporting Information (SI), Table S1). At the end of the reaction, the immobilized enzyme was separated by centrifugation (14.000 rpm, 10 min), manually collected, and stored at 5 °C for further reuse. For each case, the resulting raw reaction mixture with unreacted glycidol was directly used for the next cycloaddition step without previous purification. For time-course profiles, identical reaction vials were run simultaneously, and each point of the profile was obtained by taking aliquots of 20 μL dissolved in 580 μL of MeOH, containing 30 mM ethyl octanoate as internal standard (IS), and directly analyzed by gas chromatography (GC) (see Figures S1–S10 for details about GC analysis). The product and substrate esters content (%) were determined by the corresponding peak area balance between all of the succinate (or glutarate) species with respect to the initial succinic (or glutaric) anhydride, by using ethyl octanoate as IS. The diglycidyl succinate (or glutarate) product yield (%) was obtained from the ^1^H NMR analysis of the final reaction mixture (see Figures S15 and S16). Four different control reactions containing: (a) glycidol; (b) glycidol with Novozym 435; (c) glycidol with MS 13X; and (d) glycidol with Novozym 435 and MS 13X, incubated for 6 h at 70 °C, were carried out and analyzed by ^1^H NMR (see Figure S20)

### SILLP-Catalyzed Synthesis of Bis(cyclic carbonate) Esters by Cycloaddition of CO_2_ to Diglycidyl Moieties

The previously obtained diglycidyl ester and glycidol reaction mixture (400 μL) free of enzyme was placed into 4 mL glass vials, containing SILLPs (100 mg) and MS 13X (50 mg). Each vial was placed inside a high-pressure stainless-steel reactor (250 mL overall volume, Berghof GmbH), equipped with a magnetic stirrer, as well as temperature and pressure controls. The cycloaddition reaction was carried out by pressurizing the system with pure CO_2_ at 1 MPa for 3 h at 70 °C. At the end of the selected reaction time, the stainless-steel reactor was cooled into an ice bath for 15 min, and then slowly depressurized before opening. The reaction mixture was suspended in cold methanol (0.5 mL) and centrifuged (14.000 rpm, 10 min), and the SILLP was handled, collected, and stored for further reuse. Bis(cyclic carbonate) ester products were isolated using liquid–liquid extraction as follows: A controlled volume (0.5–1 mL) of the final reaction mixture was added to an ethyl acetate:water 1:1 (v/v) biphasic mixture (10 mL), and shaken for 10 min at room temperature. The upper organic phase, containing the target product, was then collected and dried by adding anhydrous MgSO_4_ (0.5 g). Ethyl acetate was subsequently evaporated at reduced pressure, leaving a viscous residue, which was analyzed by NMR. For NMR analysis, samples were prepared by dissolving an aliquot (50 mg) in 0.5 mL of methanol-δ_4_ (see Figure S19) (See the SI for further details on NMR analysis and quantitative determination.)

### One-Pot Synthesis of Bis(cyclic carbonate) Esters in Solvent-Free

A 4 mL open glass vial containing 2 mmol of acyl donor SA, different amounts of glycidol, Novzym-435, MS 13X, and [1-decyl-2-methylimidazolium][Cl]-based SILLP (**SILLP-4**), were placed into a stainless-steel reactor (250 mL volume, Berghoff GmbH) equipped with a magnetic stirrer, and temperature and pressure control. The reactions were carried out by pressurizing the system with pure CO_2_, or exhaust gas (15.2% CO_2_ purity), under different pressure values for a period between 6 and 24 h at a temperature ranging from 50 to 70 °C (see Table 3). At the end of the reaction, the stainless-steel reactor was cooled in an ice bath for 15 min and then slowly depressurized before opening. Finally, aliquots of 50 mg were taken from the reaction mixture and suspended in cold methanol-δ_4_ (500 μL), then centrifuged (14.000 rpm, 10 min) prior to NMR analysis (see Figures S15–S22, and yield calculation methods).

### Green Metric Parameters

The sustainability of the bis(cyclic carbonate) succinate synthesis was evaluated for the one-pot chemo-enzymatic approach using several green metric parameters, such as atom economy (AE), yield (ε), stoichiometric factor (SF), process mass intensification (PMI), reaction mass efficiency (RME), and E-Factor^36,37^ (see SI, Tables S2–S5). It should be noted that the PMI value was calculated using the ACS PMI calculator (available at https://www.acs.org/content/acs/en/greenchemistry/research-innovation/tools-for-green-chemistry.html), and the ecological-economic impact of the strategy was evaluated using the EcoScale tool (available at http://ecoscale.cheminfo.org/calculator).

## Results and Discussion

### Biocatalytic Synthesis of Diglycidyl Succinate and Diglycidyl Glutarate in Solvent-Free

The suitability of three commercial immobilized lipases (i.e., Novozym 435, Lypozyme TL IM, and Lypozyme RM IM) as catalysts for the synthesis of succinate (or glutarate) diglycidyl ester was evaluated by using different diacyl donors such as succinic anhydride (SA), glutaric anhydride (GA), dimethyl glutarate (DMG), succinic acid (Sac), glutaric acid (Gac, see Table 1), and glycidol (nucleophile acceptor) as substrates. For all cases, reaction mixtures were prepared by dissolving the acyl donor (solid) with the minimal amount of glycidol (liquid; up to 7.5–9 times mol/mol ratio) providing fully clear liquid systems after shaking at room temperature, without the presence of any additional solvent (see Table 1, entries 1–10, SRT cases). As can be seen, the suitability of the immobilized enzyme to carry out the synthesis of succinate (or glutarate) diglycidyl ester was clearly dependent on the nature of the acyl substrate. Thus, the direct esterification of glutaric (entry 1) and succinic acid (entry 2) with glycidol was not observed in these solvent-free media, even with the presence of the MS 13X dehydrating agent, which could boost the ester synthesis by shifting the reaction equilibrium through the adsorption of water byproduct.^25,36^

**Table 1:**
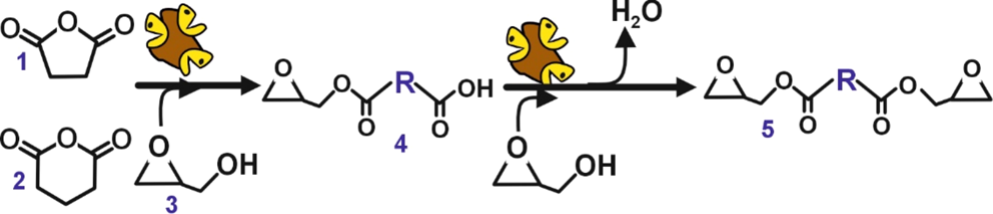
Novozym 435-Catalyzed Synthesis of Diglycidyl Succinate (or Glutarate) Esters by Transesterification and/or Esterification Reaction between Succinic Anhydride (SA), Glutaric Anhydride (GA), Dimethyl Glutarate (DMG), Succinic Acid (SAc), Glutaric Acid (GAc), and Glycidol in Solvent-Free Media at Different Temperatures^e^

entry	acyl donor (1 mmol)	glycidol (mmol)	Novozym 435 (mg)	*T* (°C)	time (h)	diester product-5^d^ (%)
**1**	GAc	7.5	40	70	6	n.d.
**2**	SAc	9.0	25	70	5	n.d.
**3**	DMG	7.5	40	70	6	4
**4**	DMS	9.0	25	70	5	6
**5**	GA	7.5	25	70	6	66
**6**	SA	9.0	25	70	5	96
**7**	GA	7.5	40	70	6	97
**8**^a^	SA	9	25	70	10	36
**9**^b^	SA	7.5	25	70	6	16
**10**^c^	GA	7.5	40	70	6	14
**11**	GA	4.0	40	50	6	43
**12**	GA	4.0	40	60	6	70
**13**	GA	4.0	40	70	6	93
**14**	SA	4.0	25	50	5	38
**15**	SA	4.0	25	60	5	71
**16**	SA	4.0	25	70	5	97

a Without MS 13X.

b Lypozyme TL IM.

c Lypozyme RM IM.

d Calculated by GC.

e See the Experimental Section for more details.

The successful synthesis of diglycidyl ester was achieved exclusively through a transesterification synthetic approach when using different ester or SA (or GA) acid derivatives, such as methyl esters or cyclic anhydrides (see Table 1, entries 3–9). When using dimethyl succinate (DMS), or dimethyl glutarate (DMG) as acyl donors, a low catalytic efficiency was observed (i.e., up to 6 and 4% diglycidyl ester, respectively, see entries 3–4), while significant synthetic outcomes (>96%) were reached when used cyclic anhydrides as acyl donors (see entries 5–8). It is worth noting that the synthesis of the diglycidyl ester product was not observed in a control reaction with MS 13X, but excluding Novozym 435, after 5 h at 70 °C. However, a slight amount of monoglycidyl ester content (8%) was detected for this reaction, likely due to the possible catalytic activity of the dehydrating agent to open the anhydride ring leading to the monoester synthesis (see Figure S10). Alternatively, the diglycidyl ester product was obtained with a low content (36%, see entry 8) for a reaction containing Novozym 435, but without MS 13X, after 10 h under vacuum (200 mbar) at 70 °C, showing the synergistic and key role of the dehydrating agent in this lipase-catalyzed transesterification/esterification reactions. These results confirm Novozym 435 as the unique catalyst for the synthesis of the diglycidyl ester product, being its synthetic activity increased by the presence of the MS 13X dehydrating agent.^30^

Other commercial immobilized lipases, such as Lypozyme TL IM and Lypozyme RM IM, showed a low synthetic activity (up to 16% diglycidyl ester content; see entries 9–10). The best results were achieved using Novozym 435 combined with MS 13X and SA (or GA) as the acyl donor substrate, reaching up to 96% diglycidyl ester content according to GC analysis (see entries 6–7). The effectiveness of SA as an acyl donor in Novozym 435-catalyzed reactions has also been reported for polyester biopolymer synthesis,^32^ the synthesis of Vitamin E succinate ester in DES (i.e., 98% yield after 6 h at 50 °C),^38^ as well as the synthesis of amphiphilic tyrosol derivatives.^39^ In the same context, Novozym 435 was shown as a more efficient biocatalyst in comparison with Lypozyme TL IM and Lypozyme RM IM, in agreement with other results previously reported for the synthesis of vinyl (meth)acrylate with glycidol by transesterification,^15^ as well as for the synthesis of cinnamyl propionate by direct esterification,^40^ in both IL media and solvent-free/DES processes.

To improve process sustainability by minimizing the required surplus of glycidol for dissolving the acyl donor anhydride, a novel method for preparing liquid reaction media was devised. This involved a preheating step (e.g. 10 min at 80 °C) for the SA (GA):glycidol substrates mixture that resulted in complete solubilization of the mixture at a 1:4 molar ratio of anhydride to glycidol. This step yielded liquid, viscous, and completely clear systems (DES-like systems), remaining unchanged after cooling and showing a similar GC peak profile to those reaction mixtures obtained without preheating (see SI Figures S5 and S6). As can be seen in entries 11–16 (Table 1), these viscous substrate solutions were also suitable media for the biocatalytic synthesis of diglycidyl succinate (or glutarate). The catalytic efficiency of the immobilized enzyme improved by increasing temperature (i.e., from 50 to 70 °C, entries 11–16), likely due to enhanced mass transfer toward the enzyme microenvironment resulting from decreased viscosity.^30,31^ However, the high reactivity of glycidol with increased temperatures. Thus, the ^1^H NMR analysis of control reactions carried out in the absence of anhydride revealed a significant reactivity of glycidol after its incubation with Novozym 435, with MS 13X, or a mixture of both for 6 h at 70 °C. In fact, signals were observed at 3.5–3.65 ppm, corresponding to a polyether backbone resulting from the uncontrolled self-polymerization of glycidol^41^ (see SI, Figures S20 and S22).

**Figure 2:**
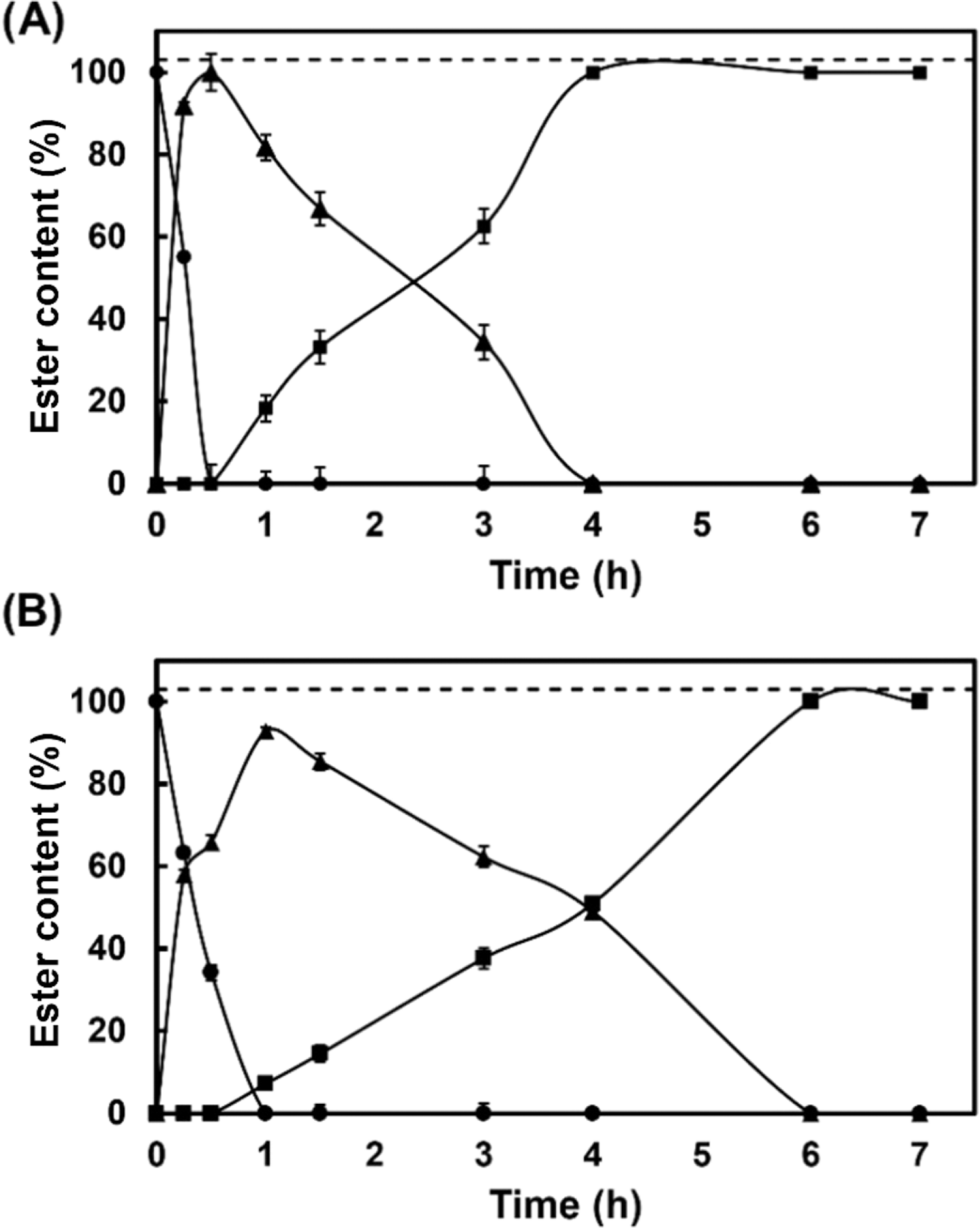
Time-course profile for the Novozym 435-catalyzed synthesis of diglycidyl succinate or diglycidyl glutarate (■, A and B, respectively) by the transesterification of SA or GA, (●) with glycidol to produce monoglycidyl succinate or glutarate (A and B), (▲), and subsequent esterification of monoglycidyl ester to obtain the diglycidyl esters as final products at 70 °C. Ester content (%) was determined by GC as described in the Experimental Section.

As a representative example, Figure 2 depicts the time-course profile for all of the detected succinate (or glutarate) derivatives during the biocatalytic synthesis of diglycidyl succinate (Figure 2A) and diglycidyl glutarate (Figure 2B), by the (trans)esterification of the corresponding anhydrides with glycidol in solvent-free at 70 °C (see Table 1, entry 6). During the first hour of the reaction, both anhydrides were fully consumed, and the only reaction product observed was the expected monoglycidyl succinate (or glutarate) intermediate, which was formed through the enzymatic transesterification reaction with ring opening of the anhydride. Furthermore, the immobilized lipase was also able to catalyze the synthesis of a second ester linkage by the direct esterification of the resulting free carboxylic group from the first reaction with another glycidol molecule, and so producing the corresponding diglycidyl ester compound. For both SA and GA, the biocatalytic transformation of anhydrides to the respective diglycidyl esters content was accomplished for approximately 97%. This transformation required either 4 h for SA, or 6 h for GA, demonstrating the high activity of the biocatalyst for this synthetic process (see Figure 2). Although the presence of the MS 13X dehydrating agent favors the immobilized enzyme-catalyzed ester synthesis with high selectivity in this nearly anhydrous reaction medium,^25,30,31^ it should be noted that the ^1^H and ^13^C NMR analysis of the final reaction mixture (entry 6, Table 1; Figure 2A) also revealed a diester derivative (glycidyl glyceryl succinate) side product that was not detected by GC analysis. This side product might have been formed by the attack of the released water molecule on a nearby epoxy group, indicating an insufficient dehydrating action of the MS 13X. As a result of this side reaction, the diglycidyl succinate target was obtained at 41 and 42% yield (see Table 1, entries 6 and 16, respectively), as determined by ^1^H NMR analysis (see SI, Figures S15 and S16 and yield calculation method).

These results show the excellent catalytic activity of Novozym 435 for the synthesis of diglycidyl esters in short reaction times and under mild and solvent-free conditions. Indeed, alternatively to classical synthetic processes based on reflux in toxic organic solvents,^23^ this straightforward and clean approach pushes again on the excellent suitability of solvent-free biocatalytic synthetic processes in restricted water environments.^15,28^ Undesired reactions, such as the oligomers resulting from the self-polymerization of glycidol or the epoxide ring opening to produce glycidyl glyceryl succinate, can be minimized by optimizing several reaction parameters, such as the amount of MS 13X, reaction time, and temperature.

### SILLP-Catalyzed Bis(cyclic carbonate)s Synthesis by Cycloaddition of CO_2_ to Glycidyl Moieties

Ionic liquids (ILs) technology has been shown as an efficient tool for the capture of CO_2_ by cycloaddition onto epoxides.^1−5,9^ By taking advantage of covalently attached ILs, also named supported ionic liquid-like phases (SILLPs),^15,29,33^ it is possible to obtain efficient catalysts with the most important features of ILs, especially with regards to its full recovery and reuse.^35,42^ In this context, the synthesis of bis(cyclic carbonate) succinyl (or glutaryl) esters by means of the cycloaddition reaction of CO_2_ to the corresponding diepoxide esters, previously obtained after the biocatalytic step, was carried out solvent-free under mild reaction conditions. The effectiveness of different SILLPs, which feature alkyl-imidazolium groups covalently attached to a styrene-divinylbenzene polymeric matrix, as catalysts for the cycloaddition reaction of CO_2_, was evaluated (see Table 2).

**Table 2:**
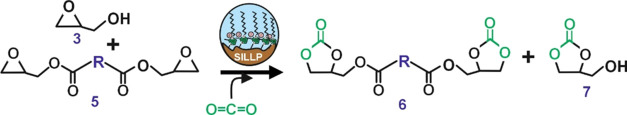
Influence of the Nature of SILLPs on the Synthesis of Bis(cyclic carbonate)s Succinate by Means of the Cycloaddition of CO_2_ to Diglycidyl Esters^a^,^c^

entry	SILLP	cation	anion	IL loading (mEq. IL g^–1^ supp)	yield-6^b^ (%)
**1**	SILLP-1	[C_1_mim]	[Cl]	5.5	14
**2**	SILLP-2	[C_4_mim]	[Cl]	4.3	18
**3**	SILLP-3	[C_10_mim]	[Cl]	1.2	15
**4**	SILLP-4	[C_10_mim]	[Cl]	4.3	28
**5**	SILLP-5	[C_10_mim]	[NTf_2_]	4.3	n.d

a [C_1_mim], 1-methyl-2- methylimidazolium; [C_4_mim], 1-butyl-2- methylimidazolium; [C_10_mim], 1-decyl-2-methylimidazolium.

b Calculated by ^1^H NMR (see the SI).

c Conditions: 400 μL of SA:glycidol (1:4 mol/mol, respectively), 100 mg SILLP, 100 mg MSX13, 3 h, 1 MPa, and 70 °C.

As can be seen, all of the assayed SILLPs were able to catalyze the targeted cycloaddition reaction on the epoxide moieties linked to succinate and provide the corresponding cyclic carbonate moieties, though relevant differences in bis(cyclic carbonate) ester yield were observed depending on the nature of the ions. Thus, the cycloaddition activity of the assayed SILLPs improved with the increase in the alkyl side chain of the imidazolium moiety (see entries 1, 2, and 4), as the ion pair becomes a weaker one making the halide more powerful toward the nucleophilic ring-opening of the oxirane. The best results were obtained for the SILLP containing [C_10_mim] as a cation, indicating that the efficiency of these catalysts can be significantly influenced by the structural characteristics of the imidazolium-based IL components.^15,43^

**Figure 3:**
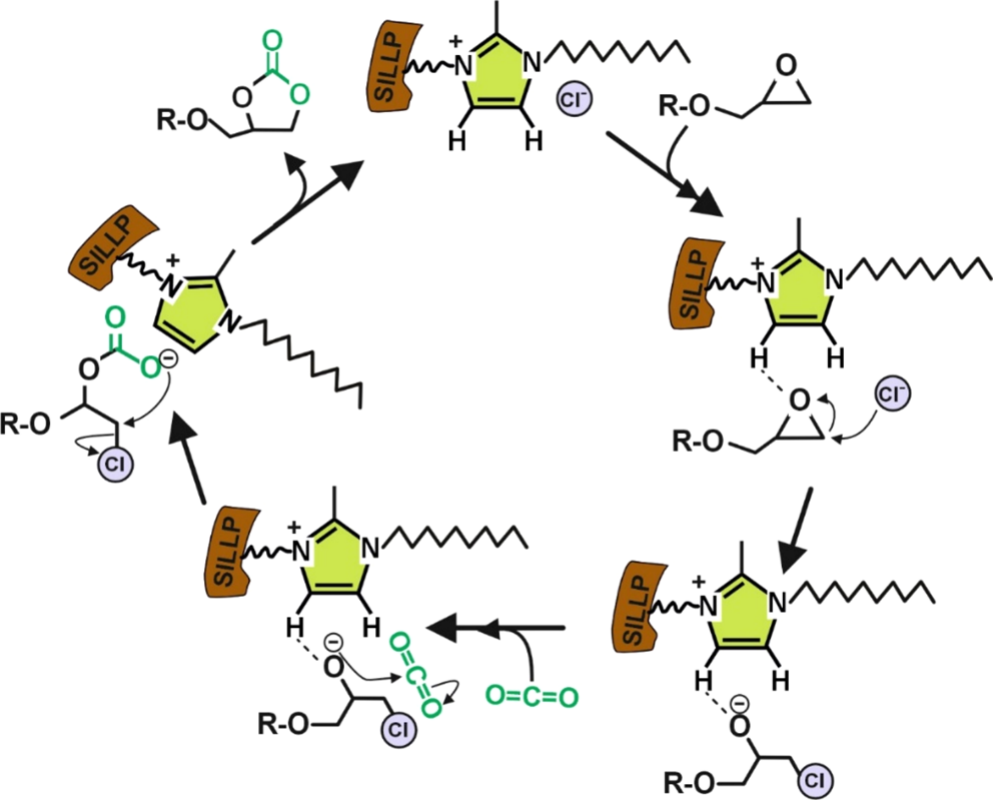
Proposed mechanism for supported imidazolium-based IL, having chloride as counteranion, as catalytic agent for ring opening of epoxide moieties and CO_2_ cycloaddition.^44−46^

Furthermore, the amount of IL loaded onto the Merrifield resin, and the specific counteranion paired with the immobilized imidazolium cation, may play critical roles in the catalytic activity of the SILLP. Thus, the increase in the IL loading from 1.2 to 4.3 mequiv per gram of polymer improves by twice the product yield (see entries 3 and 4).^33,35^ Additionally, the nature of the anion significantly influences the reaction, particularly affecting the effectiveness of the ring-opening of the epoxide group. SILLPs featuring a chloride anion demonstrated high activity for the CO_2_ cycloaddition to diglycidyl compounds, while the use of bistriflimide ([NTf_2_]) as counterion no observable activity resulted.^15^ In the same context, it has been reported how SiO_2_-supported 1-n-butyl-3-methylimidazolium halides (SBMIm·X: X = Cl, Br and I), and 1-ethyl-3-(3-(trimethoxysilyl)propyl)imidazolium halides serve as effective catalysts when utilizing a CO_2_ gas mixture from an industrial exhaust source, achieving product yields up to 100% (5 bar, 80 °C).^43^ Consequently, chloride-based SILLP-4, having the highest IL loading, was identified as the most effective catalyst for subsequent experimental investigations. Figure 3 depicts a proposed mechanism for the CO_2_ cycloaddition reaction into epoxide moieties, where the key role of ILs to increase the local CO_2_ concentration pointed out..^44^ Nucleophiles agents, such as halides (i.e., Cl^–^, Br^–^, and I^–^) catalyzed the ring-opening of the epoxide to generate an alkoxide intermediate that subsequently activates CO_2_ in the form of a hemicarbonate, which is produced upon a nucleophilic attack by the alkoxide. Finally, the system evolves to form the cyclic carbonate and regenerate the initial SILLP moiety.^45,46^

The good performance of supported IL technology in catalyzing CO_2_ cycloaddition to epoxides has been attributed to the increase in the local charge density and tunning of the orientation of the ILs near the surface.^47^ This adjustment aids in significant charge transfer, leading to surface polarization and specific adsorption of ions. Such a mechanism highlights how the physical and chemical properties of ILs can be harnessed to enhance catalytic efficiency, particularly in processes involving CO_2_ conversion. This insight into the interaction between ILs and surfaces offers a promising avenue for designing more effective catalysts for environmental and green chemistry applications, especially in the context of utilizing CO_2_ as a raw material.^48^

**Figure 4:**
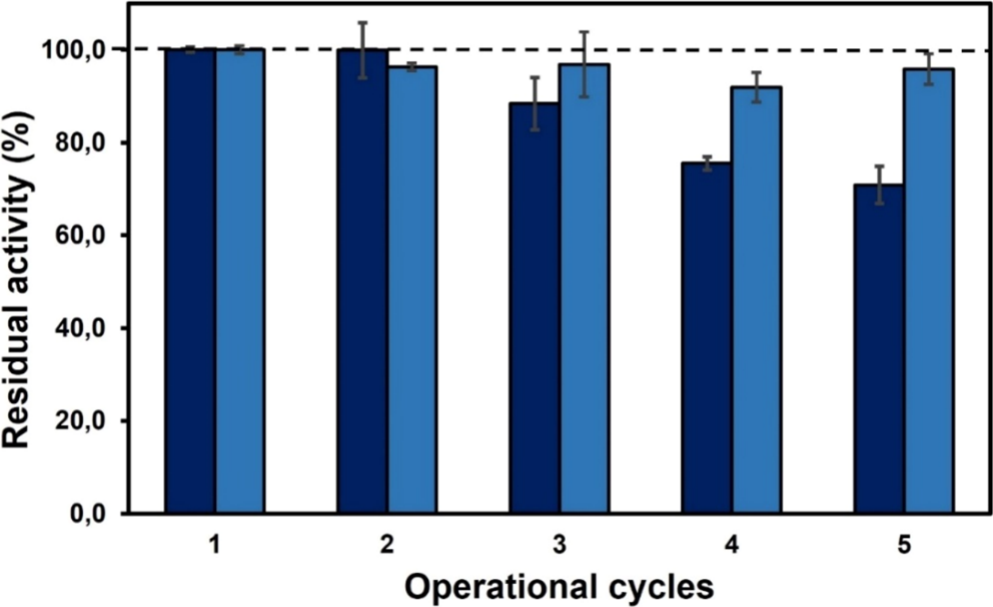
Operational stability of Novozym 435 (clear bars) for the diglycidyl succinate production (50 mg, 50 mg MS 13X, 70 °C and 6 h), and SILLP bearing [1-decyl-2-methylimidazolium] [Cl] moieties (100 mg **SILLP-4**) for the synthesis of bis(cyclic carbonate) succinate (dark bars) (1 MPa, 50 mg MS 13X, 70 °C and 3 h).

Figure 4 shows the operational stability profile for both catalysts, Novozym 435 and **SILLP-4**, for the synthesis of diglycidyl succinate and bis(cyclic carbonate) succinate, respectively, during several cycles at 70 °C. As can be seen, Novozym 435 was clearly shown to be a successfully reusable immobilized biocatalyst for carrying out the (trans)esterification of SA with glycidol in solvent-free, maintaining the residual activity practically unchanged after 5 consecutive cycles of reuse. On the other hand, the reuse of **SILLP-4** as a catalyst for the CO_2_ cycloaddition to diglycidyl succinate proved excellent for the synthesis of bis(cyclic carbonate) succinate. However, a slight decrease in residual activity was observed after each operation cycle, which was attributed to the loss of SILLP particles during the manual recovery from the viscous reaction medium.

The identification of suitable catalysts and optimal experimental conditions highlights that the synergistic combination of enzymes and SILLPs is an effective strategy for catalyzing consecutive reactions in solvent-free environments.^1,2,15^ This approach not only maintains the stability and efficiency of the catalysts but also simplifies the protocols and enhances the sustainability of the processes.

### One-Pot Chemo-Enzymatic Approach for Bis(cyclic carbonate) Esters Synthesis in Solvent-Free

**Figure 5:**
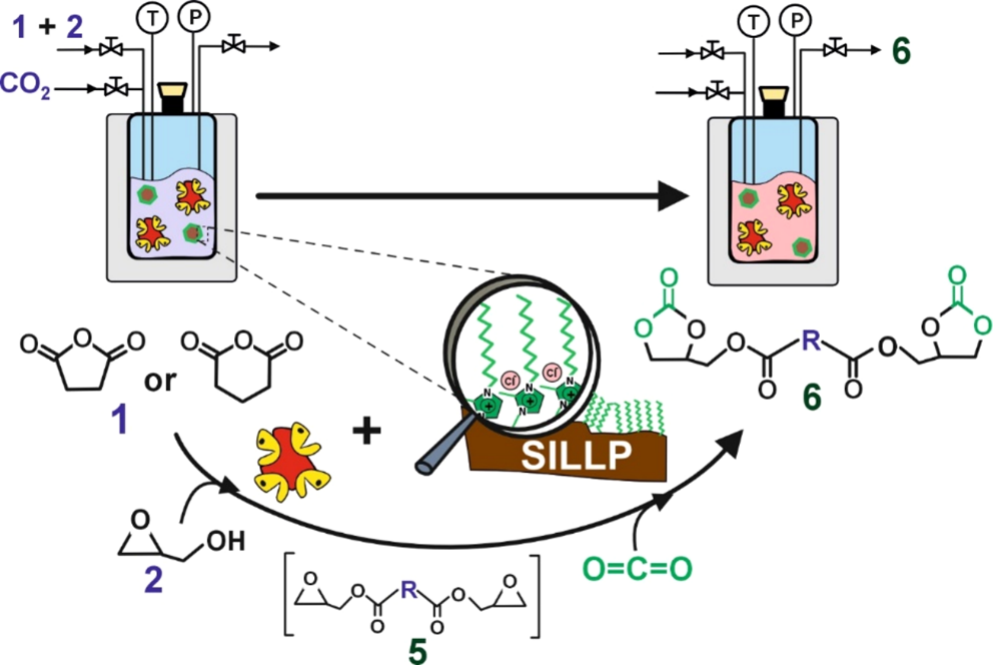
One-pot chemo-enzymatic synthesis of bis(cyclic carbonate) esters (**6**) by means of two consecutive reactions carried out by a mixture of both Novozym 435 and 1-decyl-3-methylimidazolium-based SILLP-4 catalyst particles under solvent-free conditions. SA (or GA) (**1**); glycidol (**2**).

As an effort to improve the process efficiency, the challenge of integrating the two catalytic reactions into a single step was carried out by designing a one-pot combo system under solvent-free conditions. The proposed system aims to directly convert aliphatic anhydrides into bis(cyclic carbonate) esters using the straightforward chemo-enzymatic approach, based on the direct combination of the solid cyclic anhydride (acyl donor) with glycidol as well as CO_2_, as schematized in Figure 5. By using succinic anhydride (or glutaric anhydride) as a substrate, the feasibility of this combination of chemical and enzymatic catalysts was tested not only with pure CO_2_ but also with an artificial exhaust gas containing 15.2% w/w CO_2_. To highlight the friendliness of the overall process, this work seeks to demonstrate a practical and sustainable approach to chemical synthesis by using exhaust gas as a CO_2_ source, potentially reducing reliance on pure CO_2_ supplies, and contributing to the valorization of waste gases.^1,3^

The correct balance of the reaction rates for both the enzymatic and SILLP-catalyzed steps is a key criterion for designing an effective one-pot synthetic process. To maximize the transformation of the three initial substrates (i.e., SA or GA, glycidol, and CO_2_) into a unique bis(cyclic carbonate) ester final product, the reaction parameters should be optimized to minimize the accumulation of any intermediates and the extension of uncontrolled self-polymerization reactions. Table 3 showcases the outcome of different reaction conditions based on the selection of substrate concentration (i.e., SA(or GA)/glycidol ratio), catalysts (i.e. Novozym 435 and SILLP-4), MS 13X dehydrating agent, as well as other key parameters, such as pressure, temperature, and reaction time. For all cases, the yield of the cyclic carbonate(s) ester target products was determined by ^1^H NMR (see the SI for detailed information). As can be seen in entry 1, the use of a Novozym 435/SILLP-4 combo system for a 1:7.5 (mol/mol) SA:glycidol ratio at 50 °C resulted in a 25% yield for the desired cyclic carbonate ester product. By slightly increasing the amount of biocatalyst (entry 2), or both enzyme/SILLP-4 catalysts (entry 3), a progressive improvement in product yield was observed, reaching up to 36%. However, these results did not improve with an increased amount of the MS 13X in the medium (entry 4), likely due to its participation in an uncontrolled side reaction of glycidol as described earlier. By decreasing to one-half the overall amount of substrates into the reaction media but maintaining the SILLP-4 catalyst amount present, a clear improvement in cyclic carbonate(s) ester product yield was obtained (up to 65%, entry 5). This result was also obtained when the reaction temperature increased to 55 °C (entry 6). However, increasing the temperature from 55 to 65 °C, combined with a decrease in the amount of enzyme (entry 7), reduced the final product yield by approximately 3-times (24%), which was further reduced up to 12% without the presence of the MS 13X dehydrating agent (entry 8). In the same context, a further increase in temperature to 70 °C (entries 9–15), combined with a lower SA(GA)/glycidol molar ratio (from 1:4 to 2:4.5), only offered a 12% target product yield (see entry 15).

**Table 3:**
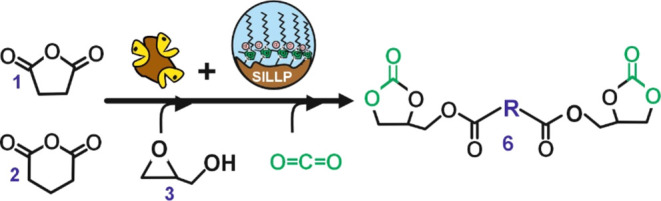
One-Pot Chemo-Enzymatic Synthesis of Bis(cyclic carbonate) Succinate from SA (of GA), Glycidol, and CO_2_ in a One-Pot Reactor Combining both Novozym 435 and SILLP-4 Catalysts under Solvent-Free Conditions

entry	acyl donor (2 mmol)	glycidol (mmol)	N435 (mg)	SILLP-4 (mg)	MS 13X (mg)	temp (°C)	press. (MPa)	time (h)	yield^a^ (%)
**1**	SA	15	100	75	100	50	1^c^	8	25
**2**	SA	15	120	75	100	50	1^c^	8	30
**3**	SA	15	120	100	100	50	1^c^	8	36
**4**	SA	15	120	100	150	50	1^c^	8	28
**5**	SA^b^	7.5	60	100	50	50	1^c^	8	65
**6**	SA^b^	7.5	60	100	50	55	1^c^	8	64
**7**	SA	15	75	100	75	65	1^c^	8	24
**8**	SA	15	75	100	0	65	1^c^	8	12
**9**	SA	8	50	100	100	70	1^c^	6	26
**10**	SA	8	50	150	100	70	1^c^	6	17
**11**	SA	8	100	100	100	70	1^c^	6	20
**12**	SA	6	50	100	100	70	1^c^	6	15
**13**	SA	6	50	100	100	70	1^c^	8	24
**14**	SA	4.5	50	100	100	70	1^c^	8	17
**15**	GA	4.5	50	100	100	70	1^d^	6	12
**16**	SA	6	50	100	100	70	1^d^	6	12
**17**	SA	6	50	100	100	70	1^d^	24	26
**18**	SA	6	50	100	100	70	0.5^d^	24	8
**19**	GA	6	50	100	100	70	0.5^d^	24	3
**20**	SA	6	50	100	100	70	0.2^d^	24	3
**21**	SA	6	50	100	100	70	0.1^d^	24	2

a Calculated by ^1^H NMR and referred to mono and bis(cyclic carbonate) esters (see the Experimental Section and SI for details).

b Succinic anhydride, 1 mmol.

c Pure CO_2_

d Exhaust Gas (EG).

Based on these comparative experiments, it can be concluded that the maximum transformation of the three initial substrates in a bis(cyclic carbonate) ester product by the one-pot approach hinges on three key factors. First, the biocatalytic reaction producing the diglycidyl esters should be faster than the cycloaddition reaction of CO_2_ into epoxide moieties. This is crucial because the free glycerol carbonate can also be opened by Novozym 435 producing glycerol,^49^ and besides the esterification reaction between glycerol carbonate with carboxylic acids needs longer reaction times (e.g., up to 3 days for the case of sebacic acid and glycerol carbonate at a 1:100 mol/mol ratio).^25^ Second, water molecules that resulted as a byproduct from the enzymatic esterification should be efficiently removed from the reaction medium, for which MS 13X was shown as a useful tool.

Finally, although temperature enhances catalysts action, higher temperatures and prolonged reaction times can lead to increased uncontrolled self-polymerization reactions between all of the components of the reaction medium (see Figure S20).^41^ Therefore, it is crucial to use moderate temperatures for this chemo-enzymatic catalytic process (e.g., approx.. 50 °C, see entry 3), and to prepare the substrate mixture at the lowest molar ratio that ensures full solubilization at room temperature (i.e., 1:7.5 mol/mol SA:glycidol ratio). The ^1^H and ^13^C NMR characterization of the bis(cyclic carbonate) succinate product can be found in Figure S19 (see the SI).

The use of an exhaust gas as a direct source of CO_2_ for its capture and transformation represents a significant challenge in the development of green and circular chemistry because it emphasizes the importance of this approach in fully recovering, recycling, and reusing materials.^1^ Within this context, the suitability of the Novozym 435/**SILLP-4** combo catalysts in a one-pot system was also evaluated for the synthesis of bis(cyclic carbonate) succinate by using exhaust gas with a low CO_2_ concentration of (i.e. 0.4% carbon monoxide, 0.40% methane, 15.2% carbon dioxide, and 84.0% nitrogen, entries 16–21, Table 3). Using the same protocol as that assayed for the case of pure CO_2_, the chemo-enzymatic systems also showed poor cyclic carbonate(s) ester yield (i.e. 12%, entry 16) after 6 h under the selected conditions (1 MPa, 70 °C), a result similar to that obtained for the pure CO_2_ case (15% after 6 h, entry 12). However, it should be noted that by extending reaction time to 24 h, the product yield obtained from the reaction with exhaust gas increased practically by 2-times (up to 26%, entry 17). The decrease in pressure of exhaust gas from 1 to 0.1 MPa led to a progressive reduction in the cyclic carbonate(s) ester product yield (up to 2%, see entries 18–21). This loss in product yield is attributed to both the lower CO_2_ concentration available for the cycloaddition reaction, and the long reaction times at 70 °C, which favor uncontrolled self-polymerization reactions, as reflected in NMR spectra (Figure S21).

These findings demonstrate for the first time the excellent suitability of the proposed one-pot approach for the synthesis of bis(cyclic carbonate) esters. This chemo-enzymatic process remains effective even when utilizing exhaust CO_2_ instead of pure CO_2_, achieving good product yields under mild reaction conditions (i.e., up to 77% for 1 MPa, 50 °C, 8 h). The use of an exhaust gas as a CO_2_ source represents a significant advancement toward the development of more sustainable and green chemical processes. This approach highlighted the role of chemo-enzymatic processes in contributing to circular economy principles by using biomass feedstocks and waste CO_2_, as raw materials for the synthesis of bis-cyclic carbonate molecules with potential applications.

### Green Metric Assessment

To assess the sustainability of the proposed chemo-enzymatic approach, the synthesis of bis(cyclic carbonate) succinate by one-pot was analyzed by means of different recognized green parameters, i.e., atom economy (AE), yield (ε), stoichiometric factor (SF), mass recovery parameter (MRP), reaction mass efficiency (RME), process mass intensification (PMI) and E-factor parameters (see SI, Table S2 for each metric parameter formula).^36^ The AE, 1/SF and ε parameters provide information about the reactivity of substrates and atoms incorporated into the desired products. The MRP informs about the recyclability of the reaction species (or the contributions to wastes), whereas the RME comprises all of them, being a global indicator of sustainability. All of these parameters range from 0 to 1, being the sustainability improved with values closer to 1.^37^ On the contrary, the PMI and E-factor parameters are used as waste quantification criteria, with the lowest values being desired (see Table S2 for calculation mode). Furthermore, the EcoScale tool was used to extend the sustainable analysis to other issues like the energy expense, the process cost, and/or toxicity of reagents by introducing different penalties to an initial value of 100% which corresponds to the maximum sustainability..^50^

Table 4 shows the scores of these green metric parameters obtained for the bis(cyclic carbonate) succinate synthesis by the one-pot approach, as well as two other reported strategies for the synthesis of bis(cyclic carbonate)s based on two-step,^23^ or one-pot,^25^ approaches, which were selected as a framework for comparison (see Table S3 for reaction conditions).

**Table 4:** Green Metrics Evaluation of the Synthesis of Bis(cyclic)carbonates Performed by Different Approaches

entry	this work	Wunschik et al.^25^	Blazek et al.^23^ first step	Blazek et al.^23^ second step
ε	0.65	1.00	0.16	0.28
AE	0.95	0.92	0.83	1.00
1/SF	0.38	0.04	0.82	1.00
MRP	0.54	0.51	0.57	0.58
RME	0.12	0.02	0.06	0.16
PMI	3.7	30.20	11.20	6.30
E-factor	2.7	29.20	10.20	5.30
EcoScale	73	92.00	25.00	48.00

For this sustainable assessment, the reaction conditions and results obtained from entry 5 (Table 3) were selected because of both the best product yield (65%) and the lowest assayed temperature (8 h, 50 °C). As can be seen, the AE parameter of this proposed chemo-enzymatic approach is very high (0.95), as a result of the direct incorporation of all of the atoms from the substrates into the target bis(cyclic carbonate) ester product, with only the loss of one water molecule. However, the excess of glycidol negatively impacts the 1/SF parameter, which ideally should also be close to one. Consequently, the unreacted glycidol undergoes CO_2_ cycloaddition, leading to the synthesis of glycerol carbonate. As a nondesired product, glycerol carbonate is included in the mass of waste, which in turn reduces the values of MRP and RME. However, glycerol carbonate is a value building block in NIPU synthesis, and it should not be considered as a real waste.^20,21^

In this context, Wunschik et al.^25^ described a solvent-free lipase-catalyzed direct esterification of sebacic acid with glycerol carbonate at a 1:100 mol/mol ratio as a suitable liquid reaction media that favors the shift of the esterification equilibrium toward the synthesis (see Table S3). This approach offers the highest yield but is achieved at the expense of a great amount of glycerol carbonate, which contributes negatively to wastes involved parameters (i.e., 1/SF, MRP, RME). Alternatively, Blazek et al.^23^ proposed a two-step catalytic approach, corresponding the first one to the epoxidation of a biobased polyether polyol (PO3G, Mw 250 Da.) with epichlorohydrin catalyzed by BF_3_·Et_2_O. After a complex purification protocol of the diglycidyl ether by using NaOH and ethyl acetate, the second catalytic step of this approach concerns the CO_2_ cycloaddition on the epoxide moieties catalyzed by tetrabutylammonium bromide (TBAB). This latter approach has a better stoichiometric factor (1/SF), but the low yield of both catalytic steps also contributed to waste generation. In any case, as the RME comprises all of the above metrics, it can be used as the most representative parameter for comparison, obtaining a clearly favorable score for the chemo-enzymatic approach here proposed.

Other relevant green parameters, such as the PMI, from which the E-factor can be extrapolated, also corroborated this overall analysis. The PMI reveals the source of wastes, being identified with nonreacted substrates and auxiliary reagents for these solvent-free approaches. In this context, the chemo-enzymatic strategy here proposed provides the lowest PMI (3.7) and hence the lower E-factor (2.7) as well.

With respect to the EcoScale, a value of 73 was obtained for this work, being the maximum penalty assigned to product yield (see SI Table S5 for the list of penalties assigned). For the two-step chemical approach,^23^ several issues, such as low yield, prizes, toxicity of epichlorohydrin, etc. have important relevance in penalties. The best qualification (92 over 100) was obtained for the enzymatic esterification of sebacic acid with glycerol carbonate (1:100 mol/mol), despite the great waste of glycerol carbonate of the approach and the low reaction rate that needs 3 days for its completion. The EcoScale parameter is an interesting tool to obtain a preliminary view of the Life Cycle Assessment of a process, provided that it is used in combination with other green metric parameters to counterbalance its weaknesses.

## Conclusions

Carbon dioxide emissions are among the most significant waste products in our society, with harmful effects on the environment. However, CO_2_ is also inherently linked to our vital activities such as respiration. As long as there is life, CO_2_ production will continue, making it essential to develop strategies for its capture and transformation. This work demonstrates the feasibility of a chemo-enzymatic green approach for the synthesis of bis(cyclic carbonate) esters from a mixture of glycidol CO_2_ and organic anhydrides in solvent-free media. By using biobased resources, this sustainable technology produces valuable precursors for manufacturing biodegradable nonisocyanate polyurethanes (NIPUs) and adhesives. The synergistic combination of biocatalytic and ILs technologies as heterogeneous catalysts enabled the transformation of substrates to cyclic carbonate(s) ester products with up to 65% yield in solvent-free media after 8 h at 50 °C, encouraging further studies to develop a flow reaction process. The sustainable nature of this approach is also supported by the suitability of using exhaust gas instead of pure CO_2_ as substrate, and the favorable scores obtained in various green metrics parameters (e.g., PMI, E-factor, and EcoScale). This work highlights the immense potential of incorporating green chemistry principles into practical applications, paving the way for producing valuable chemical products through eco-friendly processes.

## Supporting Information Available

GC-MS of the diglycidyl esters and ^13^C and ^1^H NMR analyses of the (bis)cyclic carbonate esters (PDF)

## Abbreviations

NIPU: nonisocyanate polyurethane
ILs: ionic liquids
SILLPs: supported ionic liquid-like phases
GA: glutaric anhydride
SA: succinic anhydride
GAc: glutaric acid
SAc: succinic acid
G: glycidol
GC: glycerol carbonate
DMG: dimethyl glutarate
DMS: dimethyl succinate
IS: internal standard
AE: atom economy
ε: yield
SF: stoichiometric factor
MRP: mass recovery parameter
RME: reaction mass efficiency
PMI: process mass intensification
TBAB: tetrabutylammonium bromide
