# Supplementary material for: A Green Chemo-Enzymatic Approach for CO2 Capture and Transformation into Bis(cyclic carbonate) Esters in Solvent-Free Media
Source: ACS Sustain Chem Eng. 2024 Oct 2;12(41):15033–43. doi: 10.1021/acssuschemeng.4c04102 (PMC11481583; doi:10.1021/acssuschemeng.4c04102)
Supplement: Supplementary file 1 — sc4c04102_si_001.pdf [file sc4c04102_si_001.pdf]

SUPPORTING INFORMATION.

A green chemo-enzymatic approach for CO_2 capture and transformation into bis(cyclic carbonate) esters in solvent-free media.

Rocio Villa, [1*] Francisco J. Ruiz, [1] Francisco Velasco, [1] Susana Nieto, [1] Raul Porcar, [2, 3] Eduardo Garcia-Verdugo, [2] Pedro Lozano [1*]

[1] Departamento de Bioquimica y Biologia Molecular B e Inmunologia. Facultad de Quimica, Universidad de Murcia, E-30100 Murcia, Spain

[2] Departamento de Quimica Organica e Inorganica, Universidad Jaime I, E-12071, Castellon, Spain

[3] Departamento de Química Orgánica y Bio-orgánica, Facultad de Ciencias, Universidad Nacional de Educación a Distancia, UNED, Avda. Esparta, 28232-Las Rozas, Madrid, Spain

* Corresponding autor: plozanor@um.es

Contents

1. Influence of the dehydrating agent on the biocatalytic synthesis of diglycidyl ester.

2. Control experiments.

3. GC analysis.

4. GC-MS analysis.

5. NMR analysis.

6. Green metrics parameters.

Tables

Table S1. Obtained catalytic efficiency for the synthesis of diglycidyl succinate using different dehydrating agents.

Table S2. List of Green Metric Parameters and formulas.

Table S3. Reaction conditions for the synthesis of bis(cyclic carbonates) performed by different approaches.

Table S4. Relation of products and waste produced in each strategy selected for the sustainable assessment.

Table S5. List of penalties assigned in each category of the EcoScale. The penalties are subtracted from an initial value of 100.

Figures

Figure S1. GC chromatogram of methanol containing 30 mM of ethyl octanoate as IS.

Figure S2. GC chromatogram of succinic anhydride standard.

Figure S3. GC chromatogram of glutaric anhydride standard.

Figure S4. GC chromatogram of glycidol standard.

Figure S5. GC chromatogram of a 1:4 mol/mol, glutaric anhydride:glyicidol mixture, prepared by shaking after preheating treatment (10 min, 80 °C).

Figure S6. GC chromatogram of a 1:4 mol/mol, succinic anhydride:glyicidol mixture, prepared by shaking after preheating treatment (10 min, 80 °C).

Figure S7. GC chromatogram obtained at initial reaction time of succinic anhydride:glycidol at molar ratio 1:9 (mol/mol).

Figure S8. GC chromatogram obtained after 2 h 30 min of biocatalytic reaction using succinic anhydride:glycidol at molar ratio 1:9 (mol/mol), 25 mg of Novozym 435 and 40 mg of MS 13X at 70 ºC.

Figure S9. GC chromatogram obtained after 5 h of biocatalytic reaction using succinic anhydride:glycidol at molar ratio 1:9 (mol/mol), 25 mg of Novozym 435 and 40 mg of MS 13X at 70 ºC

Figure S10. GC chromatogram of the mixture glutaric anhydride:glycidol at molar ratio 1:7.5, (mol/mol), obtained after control reaction with 50 mg of MS 13X for 5 hours at 70 ºC.

Figure S11. Experimental GC-MS spectrum of the monoglycidyl glutarate.

Figure S12. Experimental GC-MS spectrum of the diglycidyl glutarate.

Figure S13. Experimental GC-MS spectrum of the monoglycidyl succinate.

Figure S14. Experimental GC-MS spectrum of the diglycidyl succinate.

Figure S15. ^{1}H-NMR spectrum of the reaction mixture containing diglycidyl succinate and glycidol after the biocatalytic reaction using succinic anhydride:glycidol at molar ratio 1:9 (mol/mol), respectively (Table 1, entry 6).

Figure S16. ^{1}H-NMR spectrum of the reaction mixture containing diglycidyl succinate and glycidol after the biocatalytic reaction using succinic anhydride:glycidol at molar ratio 1:4 (mol/mol), respectively (Table 1, entry 16).

Figure S17. ^{1}H-NMR spectrum of the reaction mixture containing bis(cyclic carbonate) succinate and glycerol carbonate after the one-pot approach using succinic anhydride:glycidol at molar ratio 1:4 (mol/mol), respectively (Table 3, entry 9).

Figure S18. ^{1}H-NMR spectrum of the reaction mixture containing bis(cyclic carbonate) succinate and glycerol carbonate before isolation of the products.

Figure S19. ^{1}H-NMR (**A**) and ^{13}C-NMR (**B**) spectra of the isolated bis(cyclic carbonate) succinate and glycerol carbonate products after liquid-liquid extraction from the mixture with water and ethyl acetate, dried, and concentrated at reduced pressure.

Figure S20. Region corresponding to the H-atoms (2.4-4.0 ppm) of the (1) glycidol standard, and spectrum obtained after control reactions: (2) glycidol; (3) glycidol with Novozym 435; (4) glycidol with MS 13X; and (5) glycidol with Novozym 435 and MS 13X, after incubation at 70 ºC, 200 rpm and 6 hours.

Figure S21. Region corresponding to the H-atoms (2.4-5.2 ppm) of the one pot reactions described in Table 3 entries 17, 18, 20 and 21, spectra 1-4, respectively, using exhaust gas as CO_2 source.

Figure S22. ^{1}H-NMR spectrum of the reaction mixture containing bis(cyclic carbonate) succinate and glycerol carbonate after the one-pot approach using succinic anhydride:glycidol at molar ratio 1:7.5 (mol/mol), respectively (entry 5, Table 3)

1. Influence of the dehydrating agent on the biocatalytic synthesis of diglycidyl succinate.

In different 4 mL vials, mixtures 1 mmol of acyl donor succinic anhydride and glycidol were prepared at 1:4 molar ratio. The resulting mixtures were pre-incubated for 10 minutes at 80 °C under magnetic stirring, leading to a monophasic system. For each scenario, the reaction was initiated by adding Novozym 435 (50 mg) and a 40 mg of dehydrating agent (previously activated) per mmol of acyl donor (MS13X, and anhydrous MgSO4 or $CaCl_2$) to the mixture (see Table S1). Finally, aliquots of 20 µL were dissolved in 580 µL of MeOH (30 mM ethyl octanoate as internal standard), and directly analyzed by gas chromatography.

Table S1. Obtained catalytic efficiency for the synthesis of diglycidyl succinate using different dehydrating agents.

Dehydrating agent	Conversion (%)
MS13X	96.3
$MgSO_4$ anhydrous	32.5
$CaCl_2$ anhydrous	n.d.

2. Control experiments.

Control reaction of substrates mixture after pre-heating treatment

Pre-heating (PH) control experiments were carried out by mixing 1 mmol of acyl donor (*i.e.* glutaric anhydride or succinic anhydride) and glycidol at the molar ratio 1:4. The resulting mixtures were pre-incubated for 10 minutes at 80 °C under magnetic stirring, leading to a monophasic system. The reaction mixtures were incubated at 70 ºC for 6 h. Finally, different aliquots of 20 μL were taken from the mixture, dissolved in 580 μL of MeOH (30 mM of ethyl octanoate as internal standard) before and after starting the incubation protocol, and samples were directly analyzed by GC. As representative example, **Figure S5** and **S6** depicts the GC chromatograms for the glutaric / succinic anhydride:glycidol mixture, respectively after the PH treatment.

Control reaction of substrates mixture with MS 13X

MS 13X control experiment was carried out in a 4 mL vial by mixing 1 mmol of glutaric anhydride and glycidol at the molar ratio 1:7.5 mol/mol, respectively, and 50 mg of MS 13X. The resulting mixture was incubated at 70 ºC for 6 hours. Finally, an aliquot of 20 μL were taken from the mixture, dissolved in 580 μL of MeOH (30 mM of ethyl octanoate as internal standard) and directly analyzed by GC (**Figure S10**).

Control reactions of glycidol

Four control reactions with glycidol were carried out in different 4 mL vials as follow:

- **A)** 8 mmol (535 µL) of glycidol.
- **B)** 8 mmol (535 µL) of glycidol + 50 mg Novozym 435.
- **C)** 8 mmol (535 µL) of glycidol + 80 mg MS 13X.
- **D)** 8 mmol (535 µL) of glycidol + 50 mg Novozym 435 + 80 mg MS 13X.

All the resulting mixtures were incubated at 70 ºC, 200 rpm and 6 hours. Finally, different aliquots of 20 µL were taken from each mixture, dissolved in 480 µL of methanol-δ_4 after starting the incubation protocol, and samples were directly analyzed by ^{1}H-NMR (**Figure S20**).

3. Gas Chromatography analysis

Gas chromatography analysis was performed with a GC-2010-Plus apparatus (Shimadzu Europe, Germany) equipped with a flame ionization detector (FID) and a DB-1 column (30 m × 0.25 mm × 0.25 µm, Agilent, EEUU). The substrates and products were analyzed under the following conditions: total flow carrier gas (He) at 120.4 mL min^{-1}; column flow: 2.3 mL/min; injector temperature, 230 °C; split ratio 80:1; temperature program: 60 °C, 3 min; 10 °C min^{-1}, 150 °C, 2 min; 12 °C min^{-1}, 280 °C; 5 min; detector temperature, 310 °C.

Peak retention times (min) were as follows: methanol, 2.2; glycidol, 2.6; succinic anhydride, 4.6; glutaric anhydride, 7.2; ethyl octanoate, 8.9; unknown compound present in the glycidol source, 9.8; monoglycidyl succinate, 12.7; monoglycidyl glutarate, 14.3; unknown compound present in glycidol source, 18.1; diglycidyl succinate, 19.6; diglycidyl glutarate, 20.7; glutaric acid, 11.7; succinic acid, 11.7; dimethyl glutarate, 9.4;

dimethyl succinate, 7.5. Some GC chromatogram examples for peak identification are as follow:

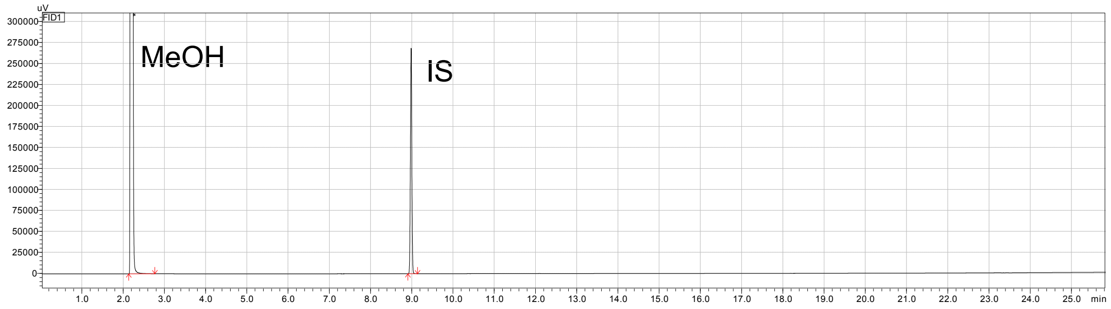

Compound	Retention time (min)	Peak area
Methanol	2.2	55895886
Ethyl octanoate (IS)	8.9	649153

Figure S1. GC chromatogram of methanol containing 30 mM of ethyl octanoate as IS.

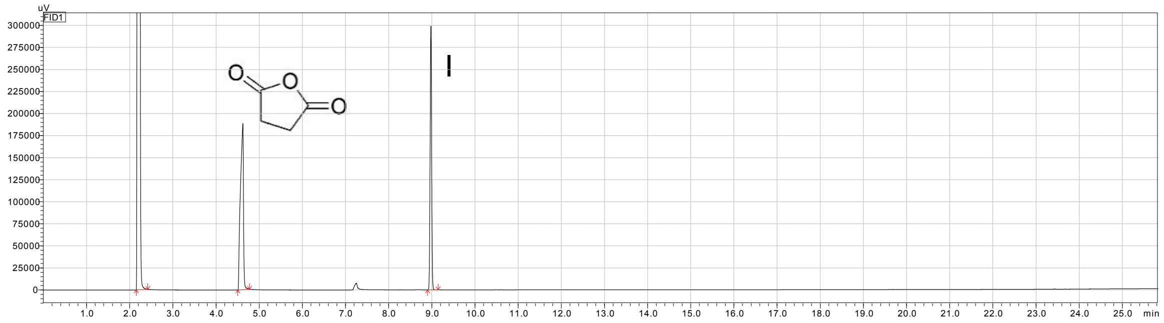

Compound	Retention time (min)	Peak area
Methanol	2.2	57450206
Succinic anhydride	4.6	188080
Ethyl octanoate (IS)	8.9	721610

Figure S2. GC chromatogram of succinic anhydride standard.

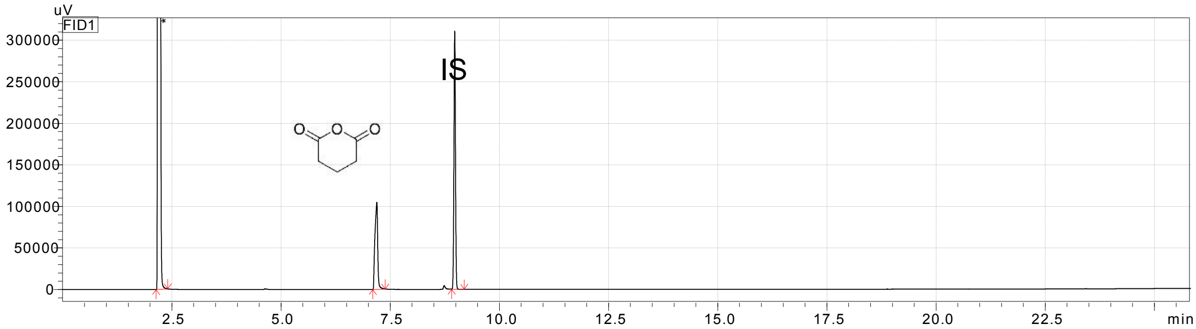

Compound	Retention time (min)	Peak Area
Methanol	2.2	59567603
Glutaric anhydride	7.2	459491
Ethyl octanoate (IS)	8.9	753153

Figure S3. GC chromatogram of glutaric anhydride standard.

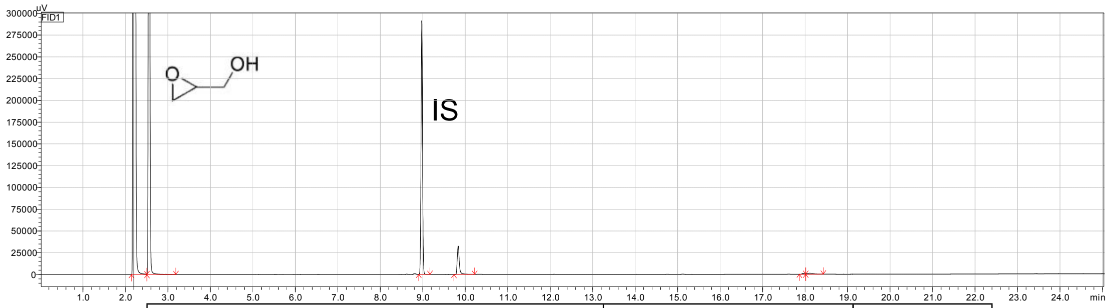

Compound	Retention time (min)	Peak area
Methanol	2.2	58602693
Glycidol	2.6	2296271
Ethyl octanoate (IS)	8.9	704212
Unknown compound in the glycidol source	9.8	108015
Unknown compound in the glycidol source	18.1	10089

Figure S4. GC chromatogram of glycidol standard.

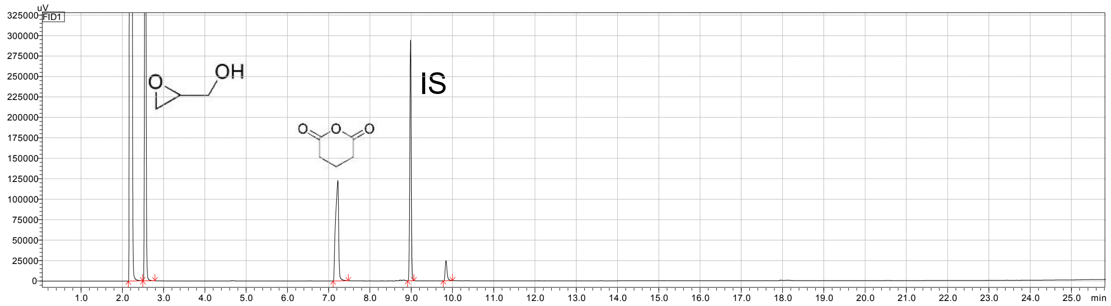

Compound	Retention time (min)	Peak area
Methanol	2.2	58046440
Glycidol	2.6	1918805
Glutaric anhydride	7.2	630374
Ethyl octanoate (IS)	8.9	710225
Unknown compound in the glycidol source	9.8	78620

Figure S5. GC chromatogram of a 1:4 mol/mol, glutaric anhydride:glyicidol mixture, prepared by shaking after preheating treatment (10 min, 80 °C).

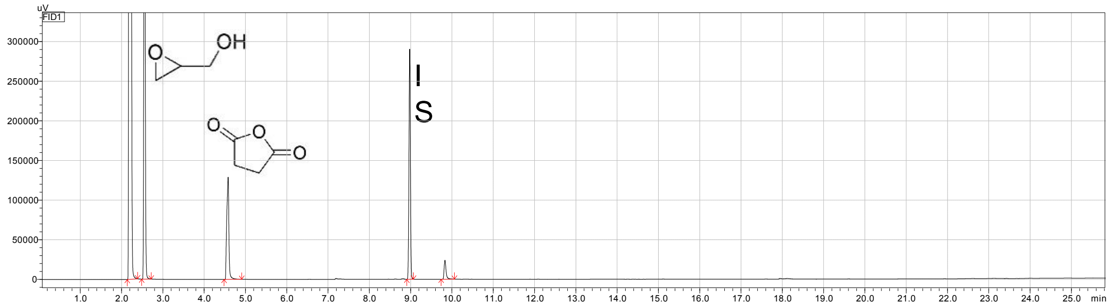

Compound	Retention time (min)	Peak area
Methanol	2.2	58382306
Glycidol	2.6	1816626
Succinic anhydride	4.6	464046
Ethyl octanoate (IS)	8.9	700000
Unknown compound in the glycidol source	9.8	78415

Figure S6. GC chromatogram of a 1:4 mol/mol, succinic anhydride:glyicidol mixture, prepared by shaking after preheating treatment (10 min, 80 °C).

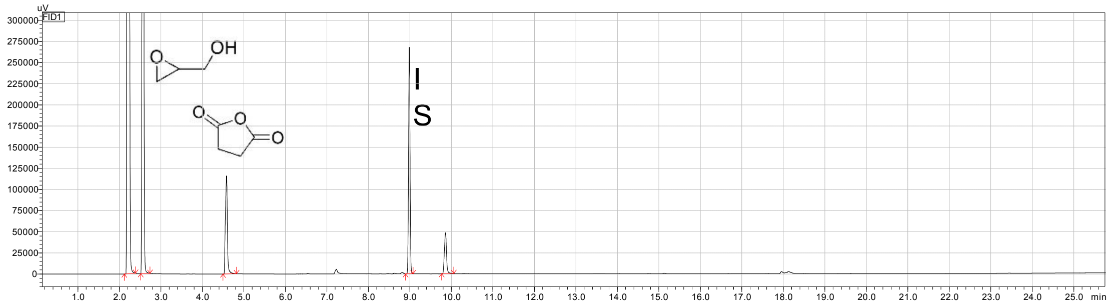

Compound	Retention time (min)	Area
Methanol	2.2	55568465
Glycidol	2.6	3552378
Succinic anhydride	4.6	385396
Ethyl octanoate (IS)	8.9	646357
Unknown compound in glycidol source	9.8	168545

Figure S7. GC chromatogram obtained at initial reaction time of succinic anhydride:glycidol at molar ratio 1:9 (mol/mol).

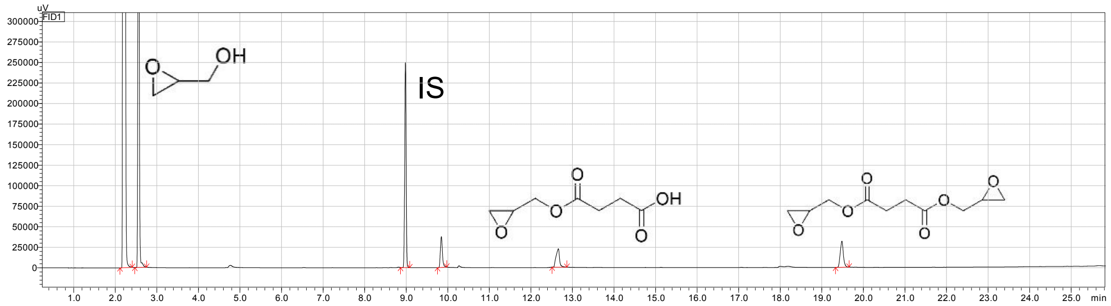

Compound	Retention time (min)	Area
Methanol	2.2	58839912
Glycidol	2.6	1588656
Ethyl octanoate (IS)	8.9	597804
Unknown compound in glycidol source	9.8	137382
Monoglycidyl succinate	12.7	127897
Diglycidyl succinate	19.6	163620

Figure S8. GC chromatogram obtained after 2 h 30 min of biocatalytic reaction using succinic anhydride:glycidol at molar ratio 1:9 (mol/mol), 25 mg of Novozym 435 and 40 mg of MS 13X at 70 ºC.

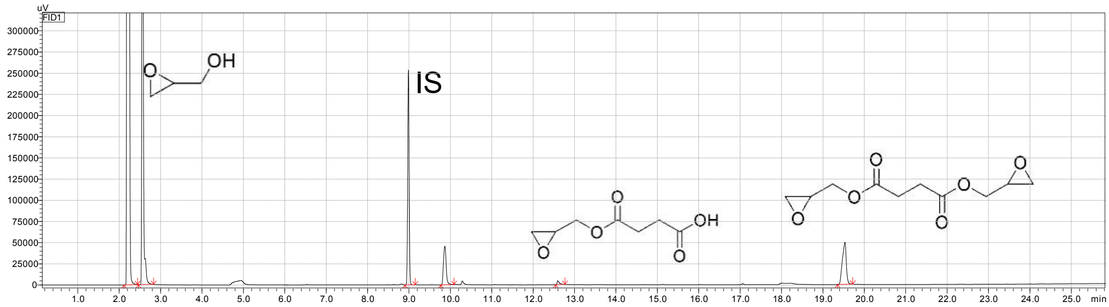

Compound	Retention time (min)	Area
Methanol	2.2	59119578
Glycidol	2.6	1394945
Ethyl octanoate (IS)	8.9	611506
Unknown compound in glycidol source	9.8	157512
Monoglycidyl succinate	12.7	16400
Diglycidyl succinate	19.5	322753

Figure S9. GC chromatogram obtained after 5 h of biocatalytic reaction using succinic anhydride:glycidol at molar ratio 1:9 (mol/mol), 25 mg of Novozym 435 and 40 mg of MS 13X at 70 ºC.

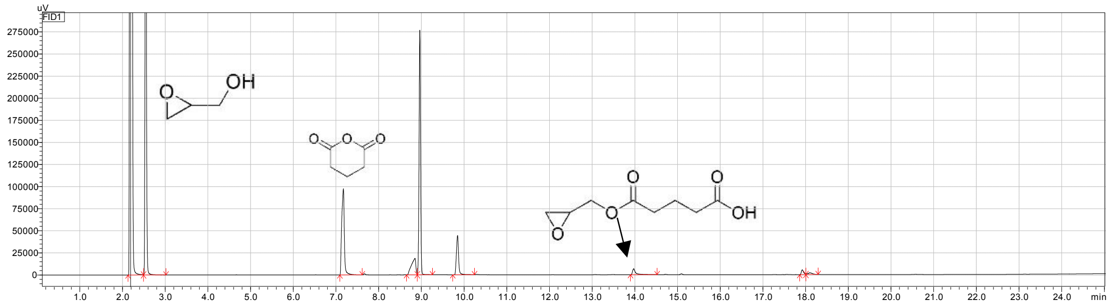

Compound	Retention time (min)	Area
Methanol	2.2	57930260
Glycidol	2.6	1334310
Glutaric anhydride	7.2	413236
Ethyl octanoate (IS)	8.9	674426
Unknown compound in glycidol source	9.8	157159
Monoglycidyl glutarate	14.0	34287
Unknown compound in glycidol source	18.1	5064

Figure S10. GC chromatogram of the mixture glutaric anhydride:glycidol at molar ratio 1:7.5, (mol/mol), obtained after control reaction with 50 mg of MS 13X for 5 hours at 70 ºC.

4. Identification of products by Gas Chromatography-Mass Spectrometry (GC-MS)

GC-MS analyses were carried out using a GC-6890 apparatus (Agilent, EEUU) coupled to a MS-5973 (Agilent, USA) system. The GC system was equipped with a HP-5MS column (30 × 0.25 µm× 0.25 µm, Agilent, USA) operated with the following parameters: carrier gas (He) at 103 mL min^{-1}; inlet split ratio: 100:1; temperature program: 40 °C, 8 min; 13 °C min^{-1}, 300 °C, 2 min; MS source ionization energy, 70 eV. The scan time was 0.5 s, covering a mass range of 400–800 amu. Monoglycidyl glutarate, retention time (rt, min): 16.3, positive ion (m/z): 57, 73, 101, 115, 129, 143, 187; monoglycidyl succinate, rt: 14.7, positive ion (m/z): 45, 73, 101, 115, 129, 157, 173; diglycidyl glutarate, rt: 22.5, positive ion (m/z): 43, 57, 101, 115, 129, 143, 171, 245; diglycidyl succinate, rt: 21.4, positive ion (m/z): 57, 73, 101, 115, 129, 157, 231.

1. *Monoglycidyl glutarate MS Spectrum (Rt: 17.1 min)*

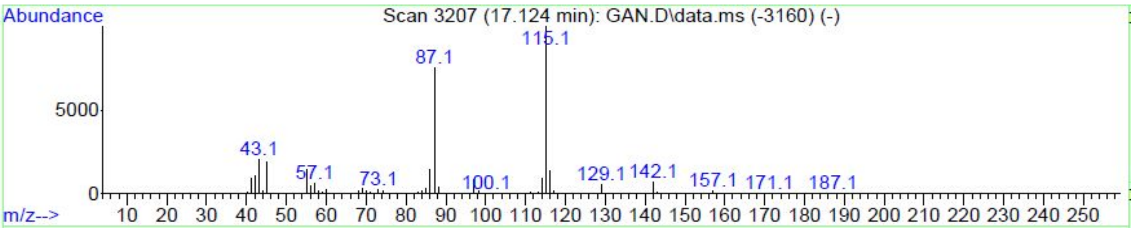

Figure S11. Experimental GC-MS spectrum of the monoglycidyl glutarate.

2. *Diglycidyl glutarate MS Spectrum (Rt: 22.7 min)*

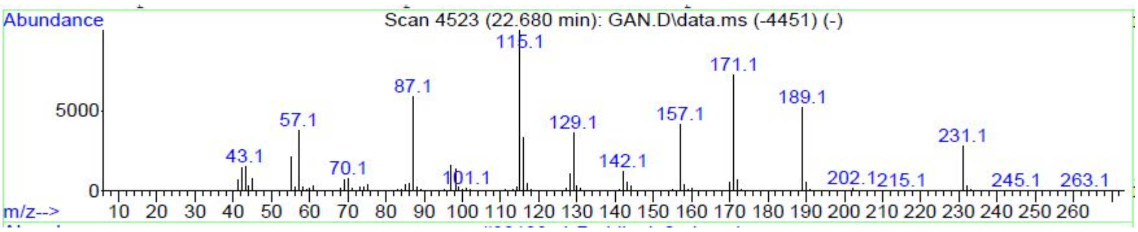

Figure S12. Experimental GC-MS spectrum of the diglycidyl glutarate.

3. *Monoglycidyl succinate MS Spectrum (Rt: 16.2 min)*

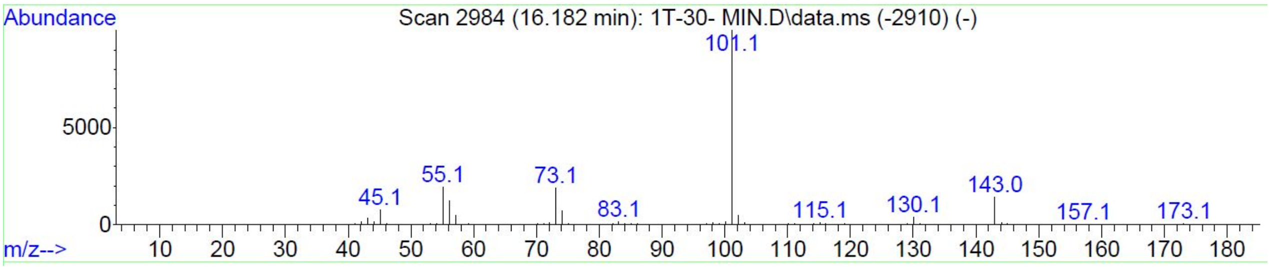

Figure S13. Experimental GC-MS spectrum of the monoglycidyl succinate.

4. *Diglycidyl succinate MS Spectrum (Rt: 21.5 min)*

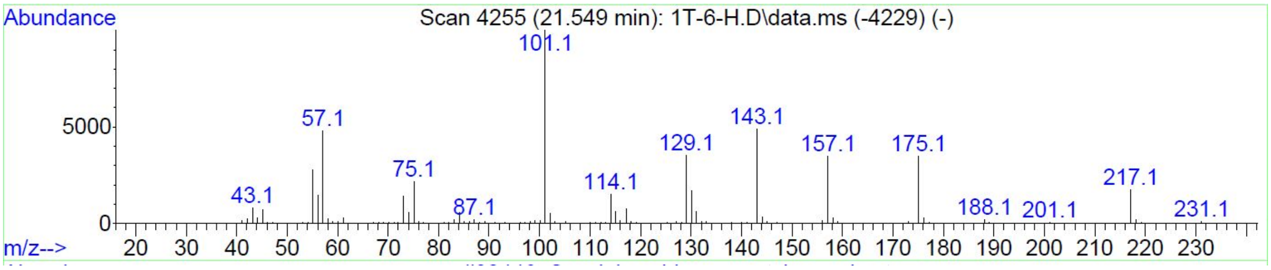

Figure S14. Experimental GC-MS spectrum of the diglycidyl succinate.

5. ^{1}H-NMR and ^{13}C-NMR analysis of bis(cyclic carbonate) glutarate or succinate

Bis(cyclic carbonate) compounds were identified by ^{1}H-NMR and ^{13}C-NMR. Experiments were performed on a Bruker Avance 400 MHz spectrometer. For NMR analysis, the longitudinal relaxation times (T1) in the sample were as follows: 380.861 ms for quantitative ^{13}C-NMR, and 509.496 ms for ^{1}H-NMR. The relaxation delay (d1) of the experiments were as follows: 5 s for quantitative ^{13}C-NMR, and 1 s for ^{1}H-NMR

Diglycidyl and cyclic carbonate(s) esters were dissolved in methanol-δ_4 (0.5 mL). The assignment of almost the totality of protons and carbons was achieved by mono-dimensional spectra.

^{1}H-NMR glycidol δ (ppm): 3.78 (dd, 1H, Ha); 3.46 (m, 1H, Ha′); 3.07 (m, 1H, Hb); 2.75 (dd, 1H, Hc); 2.62 (dd, 1H, Hc′). ^{1}H-NMR glycerol carbonate δ (ppm): 3.83 (dd, 1H, Ha); 3.63 (dd, 1H, Ha′); 4.81 (m, 1H, Hb); 4.54 (dd, 1H, Hc); 4.37 (dd, 1H, Hc′). ^{1}H-NMR diglycidyl glutarate δ (ppm): 4.42 (dd, 1H, Ha); 3.91 (dd, 1H, Ha′); 3.20 (m, 1H, Hb); 2.81 (dd, 1H, Hc); 2.64 (dd, 1H, Hc′); 2.43 (m, 4H, Hd); 1.92 (t, 4H, He). ^{1}H-NMR diglycidyl succinate δ (ppm): 4.42 (dd, 1H, Ha); 3.91 (dd, 1H, Ha′); 3.20 (m, 1H, Hb); 2.81 (dd, 1H, Hc); 2.64 (dd, 1H, Hc′); 2.67 (m, 4H, Hd). ^{1}H-NMR bis(cyclic carbonate) glutarate δ (ppm): 4.58 (dd, 1H, Ha); 4.22 (m, 1H, Ha′); 5.03 (m, 1H, Hb); 4.37 (dd, 1H, Hc); 4.32 (dd, 1H, Hc′); 2.44 (m, 4H, Hd); 1.91 (t, 4H, He). ^{1}H-NMR bis(cyclic carbonate) succinate δ (ppm): 4.58 (dd, 1H, Ha); 4.22 (m, 1H, Ha′); 5.03 (m, 1H, Hb); 4.37 (dd, 1H, Hc); 4.32 (dd, 1H, Hc′); 2.68 (m, 4H, Hd).

^{13}C -NMR glycidol δ (ppm): 63.5 (C_A), 53.3(C_B), 44.8 (C_C). ^{13}C -NMR glycerol carbonate δ (ppm): 67.3 (C_A), 78.7 (C_B), 62.2 (C_C), 157.6 (C_D). ^{13}C-NMR diglycidyl glutarate δ (ppm): 66.1 (C_A); 50.4 (C_B); 45.1 (C_C); 33.8 (C_D); 21.1 (C_E); 174.6 (C_F). ^{13}C-NMR diglycidyl succinate δ (ppm): 66.4 (C_A); 50.3 (C_B); 45.1 (C_C); 29.8 (C_D); 174.0 (C_E). ^{13}C-

NMR bis(cyclic carbonate) glutarate δ (ppm): 67.5 (C_A); 75.9 (C_B); 64.7 (C_C); 33.8 (C_D); 21.1 (C_E); 174.6 (C_F); 156.9 (C_G). ^{13}C-NMR bis(cyclic carbonate) succinate δ (ppm): 67.5 (C_A); 75.9 (C_B); 64.7 (C_C); 29.6 (C_D); 174.0 (C_E); 156.9 (C_F).

The numeration of protons and carbons of the following structure was used for the assignment:

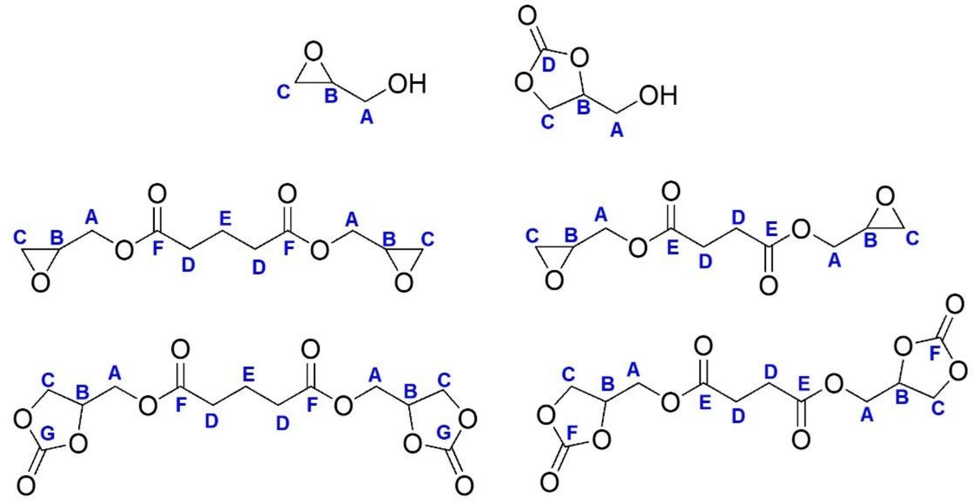

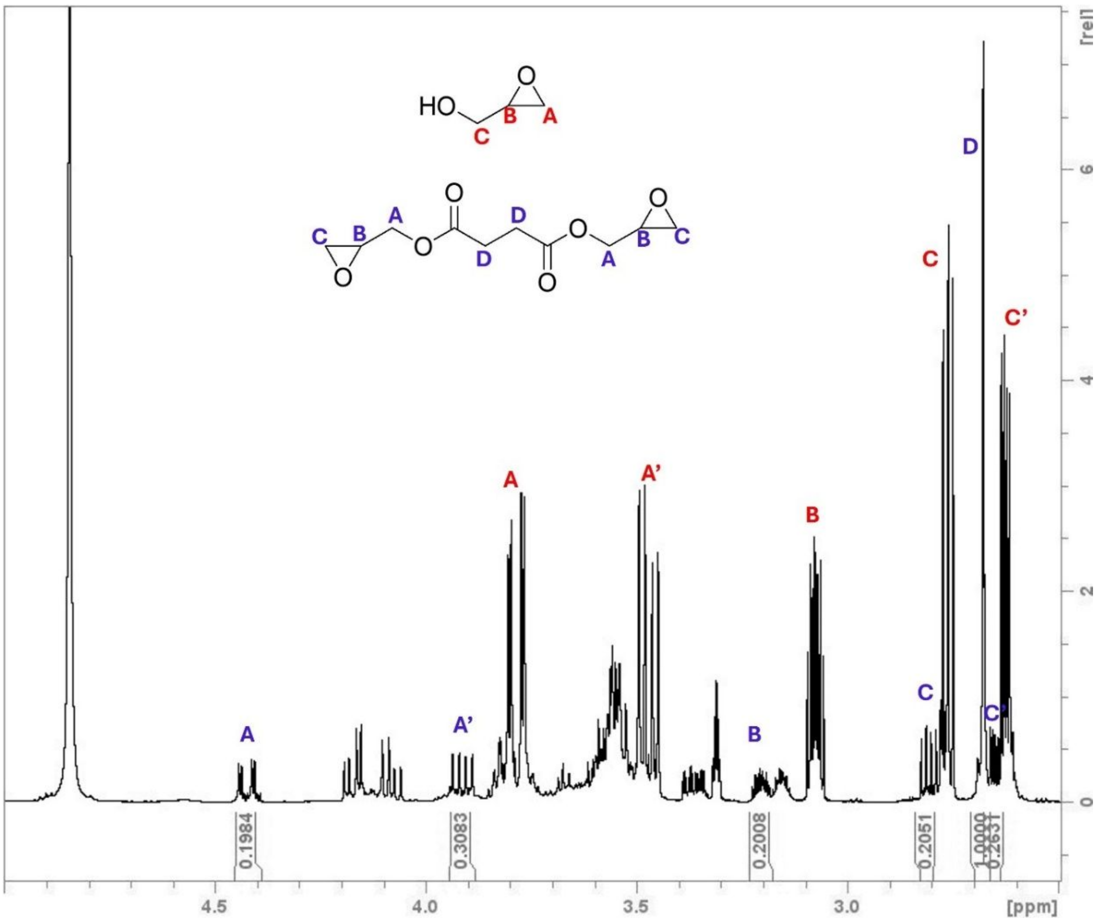

Figure S15. ^{1}H-NMR spectrum of the reaction mixture containing diglycidyl succinate and glycidol after the biocatalytic reaction using succinic anhydride:glycidol at molar ratio 1:9 (mol/mol), respectively (**Table 1**, entry **6**).

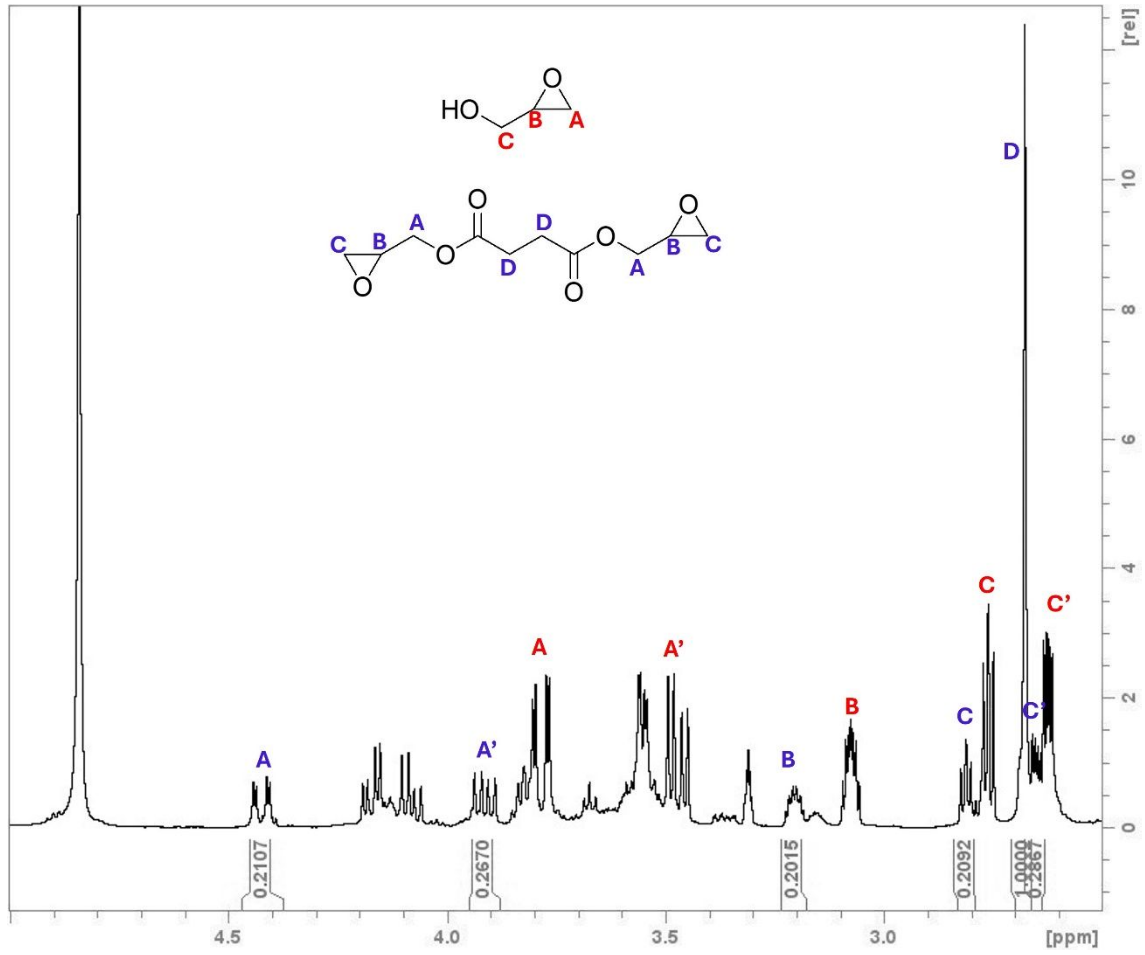

Figure S16. ^{1}H-NMR spectrum of the reaction mixture containing diglycidyl succinate and glycidol after the biocatalytic reaction using succinic anhydride:glycidol at molar ratio 1:4 (mol/mol), respectively (**Table 1**, entry **16**).

As a representative example, Figure S17 shows the ^{1}H-RMN spectrum of the final reaction mixture containing bis(cyclic carbonate) succinate and glycerol carbonate products. The following one-pot reaction system was assayed using succinic anhydride and glycidol at molar ratio 1:4 mol/mol, respectively, 50 mg N435, 100 mg SILLP-[C_{10}mim][Cl], 100 mg MS 13X, 1 MPa of CO_2, 70 °C, 6 h.

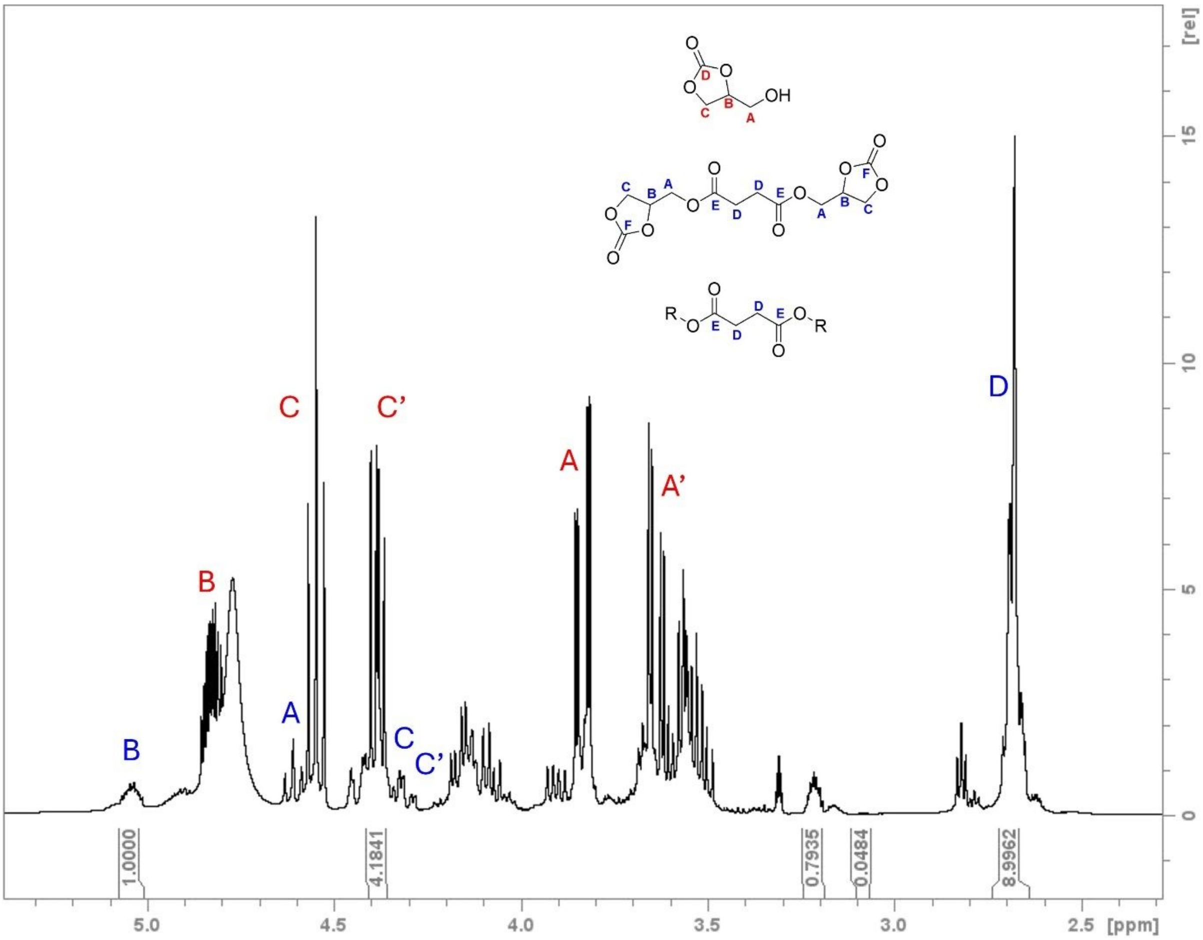

Figure S17. ^{1}H-NMR spectrum of the reaction mixture containing bis(cyclic carbonate) succinate and glycerol carbonate after the one-pot approach using succinic anhydride:glycidol at molar ratio 1:4 (mol/mol), respectively (Table 3, entry 9).

The ^{1}H and ^{13}C experiments were recorded in methanol-δ_4. As can be seen bellow, the quantitative ^{13}C-NMR spectrum of the and the bis(cyclic carbonate) succinate product after the CO_2 cycloaddition reaction before and after product isolation by means of liquid-liquid extraction with water and ethyl acetate, drying the organic phase with anhydrous magnesium phosphate, and concentrated at reduced pressure (Figure S18 and S19, respectively).

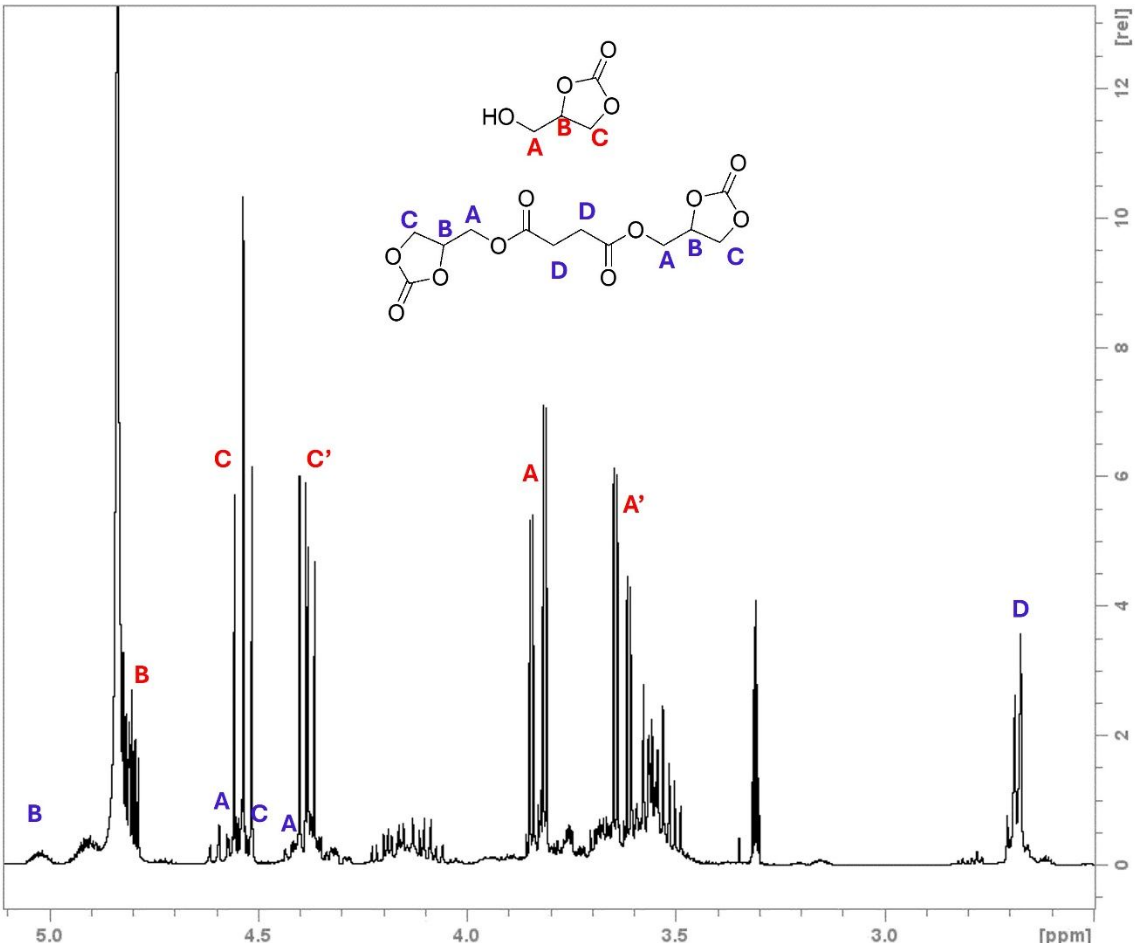

Figure S18. ^{1}H-NMR spectrum of the reaction mixture containing bis(cyclic carbonate) succinate and glycerol carbonate before isolation of the products.

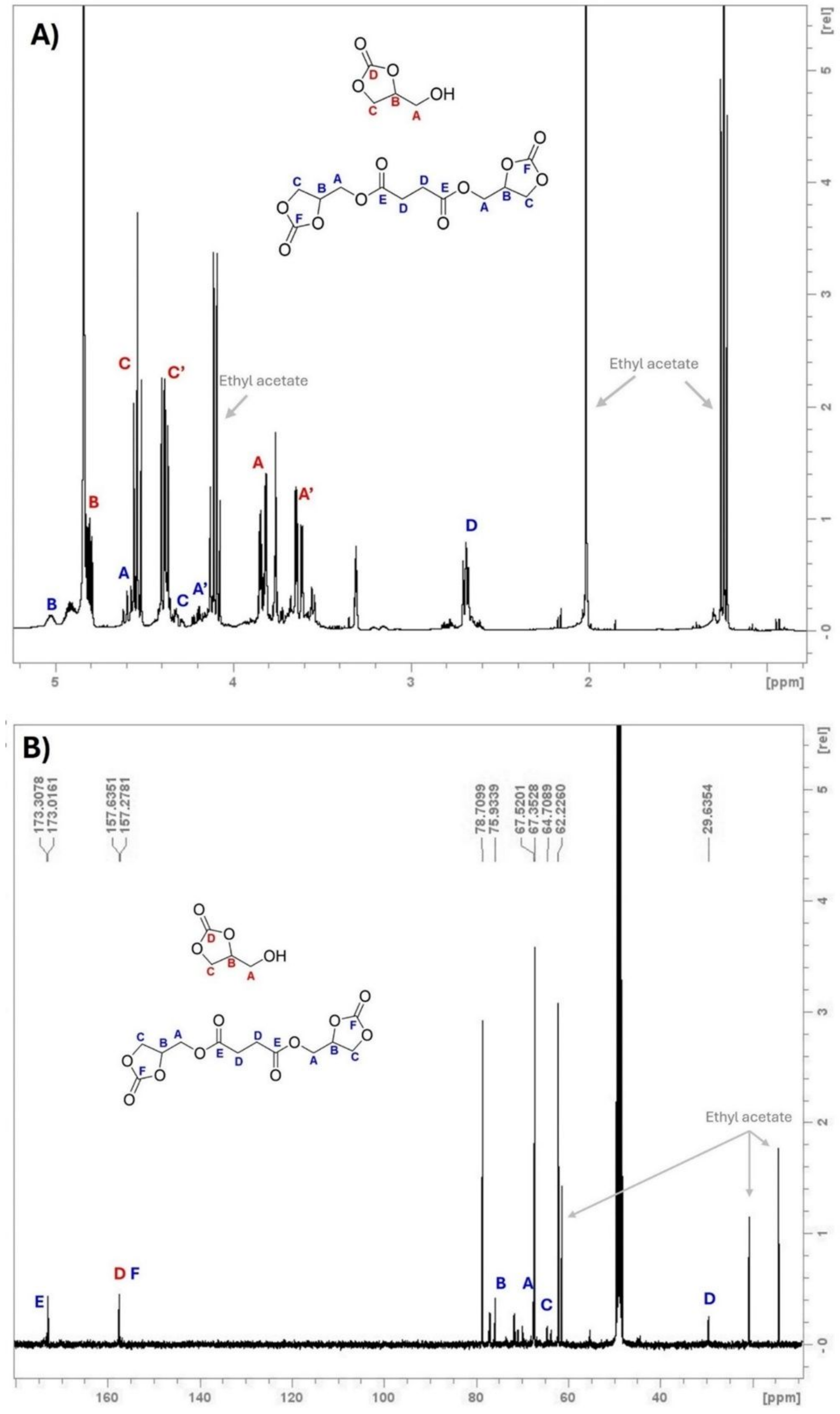

Figure S19. ^{1}H-NMR (**A**) and ^{13}C-NMR (**B**) spectra of the isolated bis(cyclic carbonate) succinate and glycerol carbonate products after liquid-liquid extraction from the mixture with water and ethyl acetate, dried, and concentrated at reduced pressure.

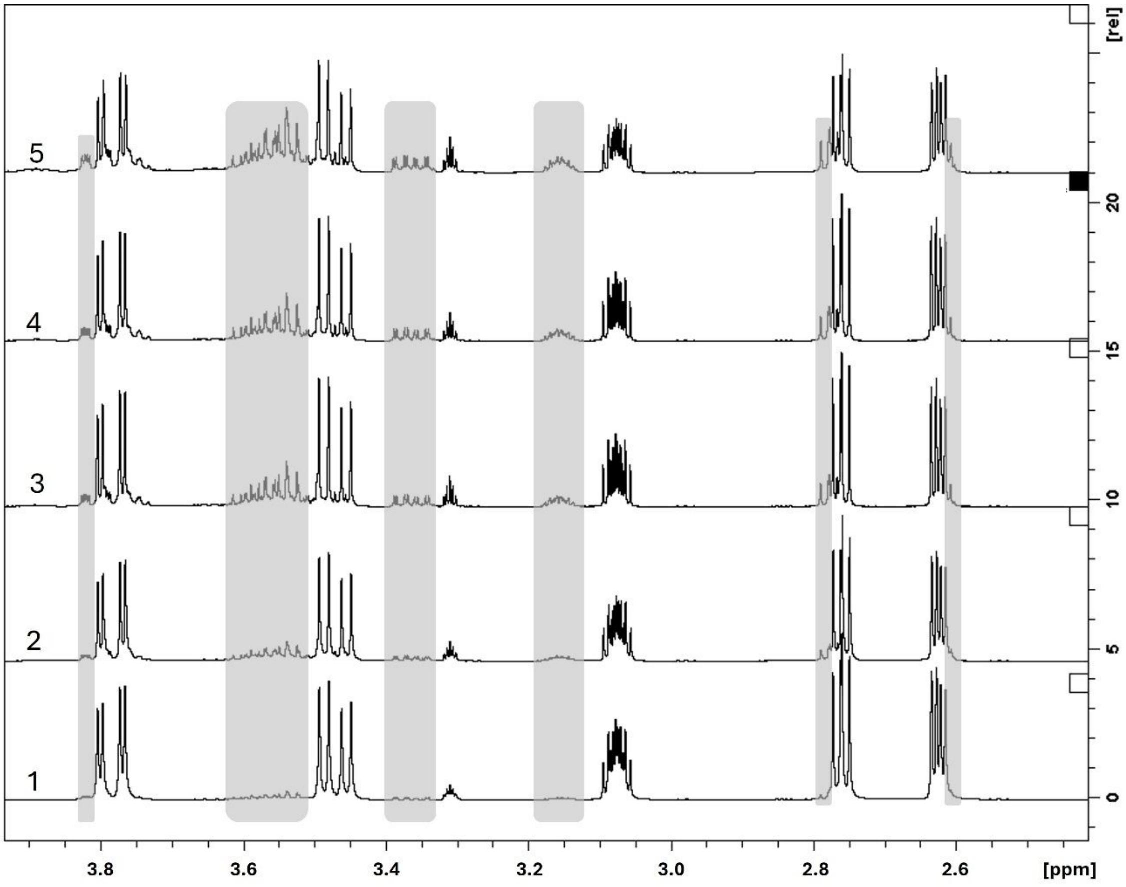

Figure S20. Region corresponding to the H-atoms (2.4-4.0 ppm) of the (**1**) glycidol standard, and spectrum obtained after control reactions: (**2**) glycidol; (**3**) glycidol with Novozym 435; (**4**) glycidol with MS 13X; and (**5**) glycidol with Novozym 435 and MS 13X, after incubation at 70 ºC, 200 rpm and 6 hours.

As representative example, Figure S21 depicts the ^{1}H-NMR comparative spectra obtained for the one-pot system (Table 3 entries 17, 18, 20 and 21) using exhaust gas as CO_2 source after reaction time. As can be observed, the polymerization of glycidol increases using lower pressure (< 1 MPa), avoiding the synthesis of bis(cyclic carbonate) succinate, as well as the synthesis of glycerol carbonate itself.

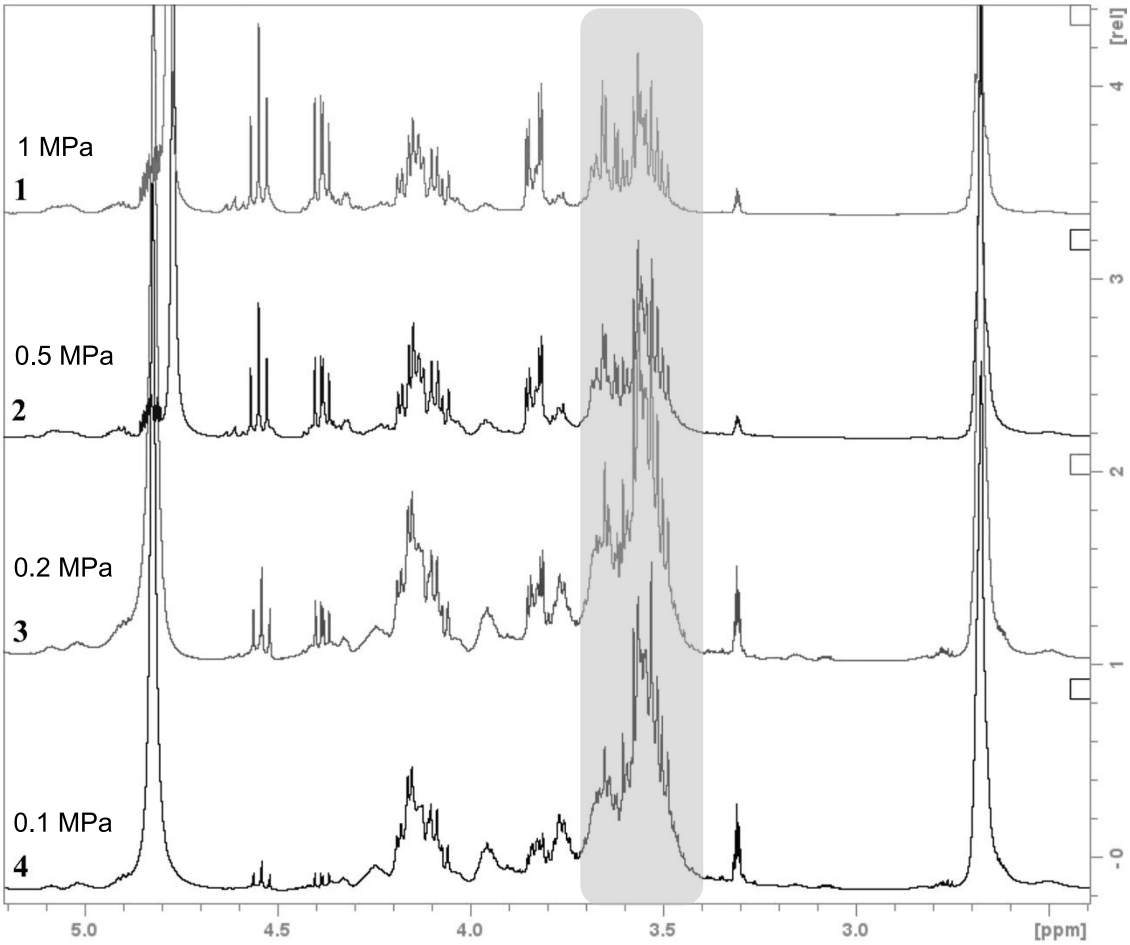

Figure S21. Region corresponding to the H-atoms (2.4-5.2 ppm) of the one pot reactions described in Table 3 entries 17, 18, 20 and 21, spectra 1-4, respectively, using exhaust gas as CO_2 source.

Diglycidyl esters yields calculation method.

The final diglycidyl ester product yield (H-Yield, %) was calculated by using ^{1}H-NMR spectrum data with the following formula:

Yield (%) = (Hc / (Hd / 2)) * 100

Where Hc corresponds to the CH proton of diglycidyl esters (2.81 ppm, succinate or glutarate) (A), and Hd are the characteristic CH_2 protons that corresponds to the succinate (B) or glutarate (C) species (2.68 ppm)

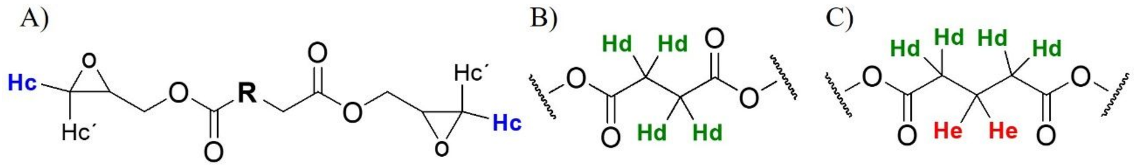

Cyclic carbonate(s) esters yield calculation method.

The final bis(cyclic carbonate) ester product yield (Yield, %) was calculated by using ^{1}H-NMR spectrum data with the following formula:

Yield (%) = (Hb / (Hd / 2)) * 100

Where Hb corresponds to the CH proton of glycerol carbonate esters (5.03 ppm, succinate or glutarate) (A), and Hd are the characteristic CH_2 protons that corresponds to the succinate (B) or glutarate (C) species (2.68 ppm).

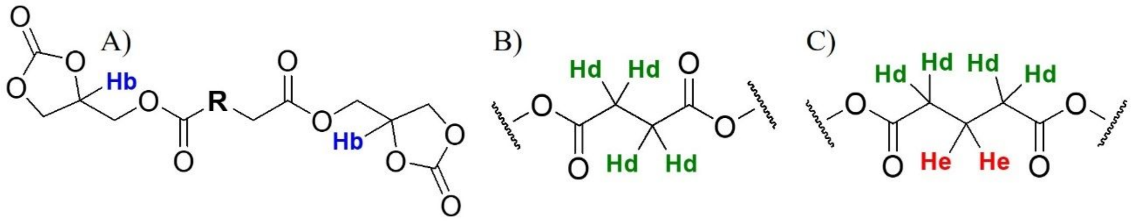

In reaction mixtures where diglycidyl esters remain unreacted, the Hd signal corresponding to the CH_2 group of the succinate (B) or glutarate (C) species (2.68 ppm) overlaps with the Hc' signal of the diglycidyl ester (2.64 ppm). To accurately determine the integration of the signal solely attributed to the CH_2 group of the succinate (B) or glutarate (C), it is necessary to subtract the contribution from overlapped proton signal of the corresponding diglycidyl ester.

This subtraction is done by using the isolated signal at 3.20 ppm, which is assignable to Hb of the diglycidyl ester. By using this approach, the moles corresponding to the CH_2 group of the total succinate (B) or glutarate (C) species can be calculated.

As a representative example (entry 5, Table 3), the yield of cyclic carbonate(s) derived from succinate is calculated as follows:

Moles of succinic ester derivatives = [Signal Integration between (2.64-2.68 ppm) – (Signal Integration of Hb of B (3.20 ppm)) / 1)]/ 2 = [3.6398 – (0.5442 / 1)]/2 = 1.5478

Moles of succinic ester carbonate = Signal Integration Hb (5.03 ppm) of A /1 = 1

Yield (%) carbonate = Moles of succinic ester carbonate / (Moles of succinic ester derivatives + Moles of succinic ester carbonate) x 100 = (1 / 1.5478) x100 = 65 %

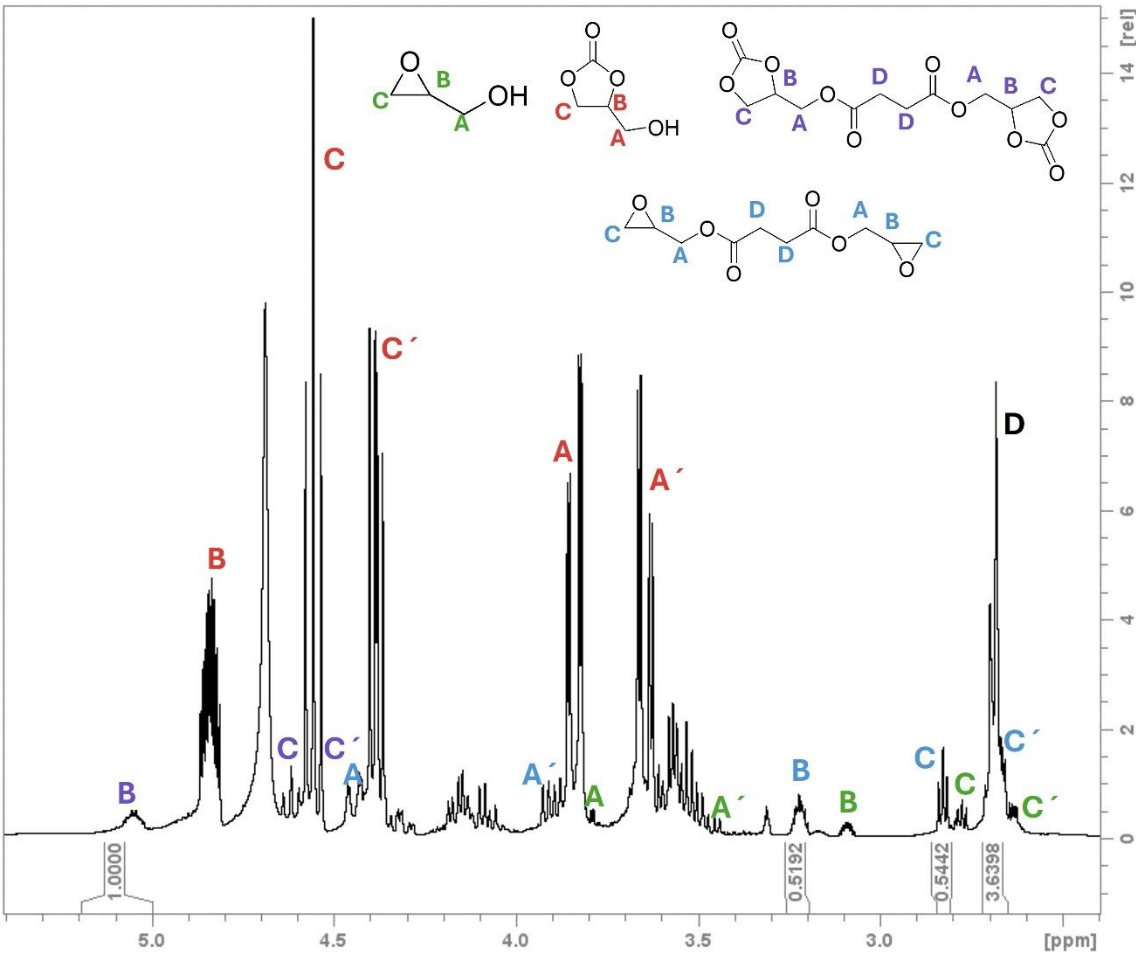

Figure S22. ^{1}H-NMR spectrum of the reaction mixture containing bis(cyclic carbonate) succinate and glycerol carbonate after the one-pot approach using succinic anhydride:glycidol at molar ratio 1:7.5 (mol/mol), respectively (entry 5, Table 3).

.

6. Green Metric Parameters

Data used to calculate the Green Metric Parameters (AE, 1/SF, ε, PMR and RME) and those introduced in the **ACS PMI Calculator** (https://www.acs.org/content/acs/en/greenchemistry/research-innovation/tools-for-green-chemistry.html), and the **EcoScale** webtool (http://ecoscale.cheminfo.org/calculator), which were used to perform the sustainability analyses, are shown in Tables S2-S6. See references [36,37] for any further detail.

Note that those recovered reagents are not considered for waste calculations.

Table S2. List of Green Metric Parameters and formulas. Mw: molecular weight; MSP: Mass of synthesized esters; MME: Maximum mass of esters; C: Catalyst (g); S: Substrates (g); W: Wastes (g). [36,37]

Parameter	Abbreviation	Calculation
**Atom Economy**	**AE**	$\mathrm{AE} = \frac{Products\ Mw}{\Sigma\ \mathrm{Reactants\ Mw}}$
**Stoichiometric Factor**	**SF**	$\mathrm{SF} = 1 + \frac{Mass\ of\ Excess\ of\ Reactives\ (g)}{Mass\ of\ Stoichiometric\ Reactives\ (g)}$
**Yield**	**ε**	$\varepsilon = \frac{\mathrm{MSE}}{\mathrm{MME}}$
**Material Recovery Parameter**	**MRP***	$\mathrm{MRP} = \frac{1}{1 + \frac{\varepsilon \cdot \mathrm{AE} \cdot (\mathrm{C}+\mathrm{S}+\mathrm{W})}{\mathrm{SF} \cdot \mathrm{MSE}}}$
**Reaction Mass Efficiency**	**RME**	$RME = \frac{\varepsilon \cdot \mathrm{AE} \cdot \mathrm{MRP}}{\mathrm{SF}}$
Process Mass Intensification	PMI	$\mathrm{PMI} = \frac{1}{AE\ \varepsilon\ \frac{1}{SF}\ \left(\frac{1}{1+AE\ \varepsilon\ \frac{1}{SF}\ \frac{(\mathrm{C+S+W})}{MSP}}\right)} = \frac{1}{RME}$
**E-factor**	**E-factor**	$E = \frac{1-\mathrm{RME}}{\mathrm{RME}}$

*All recovered reagents (solvents, catalysts, etc.) are not included in MRP calculations.

Table S3. Reaction conditions for the synthesis of bis(cyclic carbonates) performed by different approaches.

Entry	This work	Wunschik et al [25]	1st Step<br>Blazek et al [23]	2nd Step<br>Blazek et al [23]
Reaction	(Trans)esterification of Succinic anhydride with Glycidol (1:7.5 mol :mol), followed by CO_2 cycloaddition	Enzymatic esterification of sebacic acid with glycerol carbonate (1: 100 mol:mol)	Etherification of polyether polyol PO3G250 with Epichlorohydrin (1:3 mol: mol)	Cycloaddition of CO_2 to diglycidyl ether
Catalyst (g)	N435 (0.06) + SILLP (0.1)	Free CalB (0.1)	$BF_3 \cdot Et_2O$ (0.6)	TBAB (1.0)
Conditions	Incubation 50 °C, 200 rpm, 8h, 1 MPa CO_2	Stirring 72 h, RT	Incubation at 80 °C, 200 rpm, 8 h. Recovery of products: After cooling down to 50 °C, medium is washed with 50% w/w aqueous solution of NaOH added dropwise over 5 h to precipitate salts. Product extraction with ethyl acetate.	110 °C, 30 h under atmospheric pressure with a 100 ml min^{-1} CO_2 gas flow rate

Table S4. Relation of products and waste produced in each strategy selected for the sustainable assessment.

**Entry**	**SUBSTRATES (mmol)**	**Yield (%)**	**PRODUCTS (mmol)**	**Product mass (g)**	**WASTE (mmol)**	**Waste mass (g)**
One-pot This work	Succinic anhydyde (1 mmol) + Glycidol (7.5 mmol) + CO_2 (72 mmol)	65	Bis(cyclic carbonate) succinate (0.65)	0.2	Glycerol carbonate (6.22) + H_2O (0.64)	0.75
1st Step Blazek et al [23]	PO3G250 (250) + Epychlorohydrin (750)	16	Diglycidyl ether ED250 (40)	14.5	Non-reacted Epichlorohydrin (710) + HCl (80) + NaOH (30)+ catalysts (1 % wt)	100.2
2nd Step Blazek et al [23]	ED250 (260) + CO_2 (2213)	28	Bis(cyclic carbonate) DC250 (72.8)	32.8	Non-reacted ED250 (227,2) + Non-reacted CO_2 (2267,6) +Ccatalysts (0,5 % wt)	83.3
One-pot Wunschik et al [25]	Sebacic acid (1.5) + Glycerol carbonate (150)	100	Bis(cyclic carbonate) sebacate (1.5)	0.6	Glycerol carbonate (147) + H_2O (3)	17.5

*CO_2 is a waste *perse*, that is being revalorized in these approaches. Then, the excess of CO_2 is not accounted for waste mass.

Table S5. List of penalties assigned in each category of the EcoScale. The penalties are subtracted from an initial value of 100.

Entry	One-pot This work	One-pot Wunschik et al [25]	1st Step Blazek et al [23]	2nd Step Blazek et al [25]
Yield	-18	0	-42	-36
Price availability				
Succinic anhydride				
Glycidol	-3			
Diglycidyl succinate				
CO_2	-3			-5
PO3G250			-5	
Epichlorohydrin			-5	
ED250				-3
BF3EtO2				
TBAB				
NaOH			-5	
Glycerol carbonate		-5		
Safety*				
CO_2 (T)				-5
Epichlorohydrin (T)			-5	
NaOH (F, T)			-10	
Technical setup				
Common setup	0	0	0	0
Instruments for controlled addition of chemicals				
Temperature-Time				
Heating > 1h	-3		-3	-3
Room temperature < 24h		-1		
Work up and purification				
Simple filtration	0			
Adding solvent				
Removal of solvent with bp < 150 °C				
Solid phase extraction		-2		
**EcoScale score**	**73**	**92.0**	**25**	**48**
